# Secondary Metabolites in Basil, Bio-Insecticide, Inhibition Effect, and In Silico Molecular Docking against Proteolytic Enzymes of the Red Palm Weevil (*Rhynchophorus ferrugineus*)

**DOI:** 10.3390/plants11081087

**Published:** 2022-04-16

**Authors:** Hossam Moustafa Darrag, Hani Taher Almuhanna, Emadaldeen Hamad Hakami

**Affiliations:** 1 Department of Research and Training, Research and Training Station, King Faisal University, Al-Ahsa 31982, Saudi Arabia; 2 Pesticide Chemistry and Technology Department, Faculty of Agriculture, Alexandria University, Alexandria 21545, Egypt; 3 Research and Training Station, King Faisal University, Al-Ahsa 31982, Saudi Arabia; hmuhana@kfu.edu.sa (H.T.A.); ehakmi@kfu.edu.sa (E.H.H.)

**Keywords:** Biopesticides, biotic elicitor, compounds activity, docking energy, embryogenic calli, liquid media, target enzyme, somatic embryos

## Abstract

The purpose of this work was to determine the secondary metabolites generated by *O. basilicum* cell suspensions, as well as their insecticide and inhibitory activity against *R. ferrugineus*. The growth kinetics with inoculation *Verticillium dahliae* were determined and identified using LC-MS. Determination of total phenolic components (TFC), flavonoids (TF), and condensed tannins (TCT) were measured. Insecticidal activity of *O. basilicum* extract against *R. ferrugineus* (larva and adult) and proteolytic enzymes activity were assessed (in vitro and in vivo). The *O.* *basilicum* extract had an LC_50_ of 1238 µg/mL and an LD_50_ of 13.4 µg/larva. The LC_50_ of chicoric acid, ursolic acid, salvigenin, quercetin-3-O-rutinoside, rosmarinyl glucoside, and nepetoidin B demonstrated activity at an LC_50_ of 1132, 1167, 1189, 1214, 1275, and 1317 µg/mL, respectively. Chicoric acid, salvigenin, nepetoidin B, and rosmarinic acid demonstrated an LD_50_ activity of 10.23, 11.4, 11.9, and 12.4 µg/larva, respectively. The active extract of *O. basilicum* inhibited total protease, trypsin-like serine proteinases, elastase, cysteine, and metalloprotease activity with an IC_50_ (in vitro) of 119.4, 91, 102.4, 76.4, and 52.4 µg/mL, respectively. In silico studies of compounds were conducted, such as molecular docking and ADMET analysis. The study proposes using an efficient cell suspension technique to produce *O. basilicum* extract containing active secondary metabolites and accessible using as bio-insecticide.

## 1. Introduction

Date palm (*Phoenix dactylifera* L.) is an important economic crop that is commonly attacked by a variety of pests during its growth season. *Rhynchophorus ferrugineus* (Oliver) is one of the most harmful pests to date. Additionally, it is a significant pest of several palm species; it has been found to affect over 21 palm species globally [1,2,3], resulting in agricultural output losses. Larvae are considered to be one of the most difficult and dangerous stages, since they grow swiftly and infiltrate palms, killing and deforming palm fronds by feeding on the apical meristem [4,5]. Once infected, palms become vulnerable to infection by a range of insects, fungus, and pests through the tunnels made by larvae [6,7]. *Rhynchophorus ferrugineus* is a tropical insect prevalent and widely distributed in the Middle East and Mediterranean region, which encompasses North Africa and Europe. It is the most destructive of the ten species of the genus *Rhynchophorus*, which are widely distributed across the pan-tropics [8,9,10,11,12].

Numerous active ingredients (including fixed oils, volatile oils, and other compounds) have been extracted from natural resources that are used for pest control [13]. The larvicidal impact may be a result of a variety of separate components, including terpenoids, alkaloids, flavonoids, and sterols [14]. Certain naturally separated compounds, such as filiferol and extracts of *Justicia brandegeana* Cangelosi, et al., revealed larvicidal activity and have a strong biocontrol effect on the red palm weevil *R. ferrugineus* [15]. Tests in the lab have shown that *Invasive Alien* has insecticidal efficacy at controlling Rice Weevils [16]. It was shown to have a possible impact on enzymatic bioactivity and chitinase effectiveness, as well as a deleterious effect on protein synthesis, the enzymatic system, and DNA damage [3]. *Calotrapos gigantean* latex has been found to be pesticide against *R. ferrugineus*, and an inhibitor of serine protease [17]. According to previous studies, protease inhibitors are effective against a variety of biotic factors and have protective effect in plants, as, potentially, ecologically beneficial agrochemicals [18]. Monoterpene derivatives exhibited pesticidal activity, making them interesting candidates for the development of safe and environmentally acceptable agents [19]. Secondary metabolites might be utilized to monitor the red palm weevil by possible candidates for *R. ferrugineus* population control, such as geraniol, 1-octen-3-ol, and α-pinene [20]. Indeed, α-pinene, with methyl salicylate, in particular, demonstrated pheromone-disrupting properties [21]. Additionally, coumarin inhibited the expression of genes in the *R. ferrugineus* detoxifying process, suggesting that it might be employed as a control agent [22]. Additionally, picrotoxin may be used as a bioinsecticide to suppress infestations of *R. ferrugineus* [23].

*Ocimum basilicum* L. is a plant from the Lamiaceae family that has been historically grown globally owing to its major characteristics [24]. Numerous genera, including *Ocimum*, generate a variety of secondary metabolites, including phenols, terpenoids, flavonoids, and alkaloids, which provide a wide range of activity and applications, including antioxidant and anti-inflammatory properties, as well as antibacterial properties [25]. Monoterpenes, sesquiterpenes, phenylpropanoids derivatives, and flavonoids have been found in several species of the Lamiaceae family, including *O. basilicum* [26,27,28], and may be researched for potential use as bio-insecticides. In this context, plant cells and tissue cultures produce secondary metabolites in a regulated manner. Current productivity and yield levels are inadequate to fulfill the bioprocess objectives for secondary metabolite generation by plant cells [29,30,31]. Opportunities for plant-cell-based processes, new paths, and recent advancements are thoroughly evaluated.

Plant reproduction has been accomplished by the use of somatic embryogenesis and genotypes employing a variety of explants using meristematic cells [1]. Somatic embryogenesis is more effective and may be utilized to produce secondary metabolites. Previous research has been conducted to optimize plant somatic embryogenesis by manipulating culture medium ingredients such as auxins, amino acids, cytokinins, N-phenyl N’-1,2,3-thidiazol-5-ylurea (TDZ), abscisic acid, biotin, sucrose, thiamine, organic additives, and basal salt formulations [29,30,31,32]. There have been few investigations on the production of bioactive chemical synthesis in vitro cultures by plants. We extend our prior work on characterization of *O. basilicum* and *Thymus vulgaris* as eco-insecticides against *R. ferrugineus* [1]. As a result, such as thorough chemical composition of volatile compounds isolated from *O. basilicum* and *T. vulgaris* cells suspension. Furthermore, the growth kinetics of cell suspension extracts were investigated, studying the effect of incubated with *V. dahliae* and identifying chemical components using GC-MS [1,33,34,35,36].

There has recently been renewed interest in the secondary metabolites production in vitro from employing cell suspension cultures [1]. A hypothesis for this study used the cell suspension technique for produced secondary metabolites from *O. basilicum*, establishing a link between the polyphenolic and flavonoid (secondary metabolites) chemicals contained in *O. basilicum* and their usage as bio-insecticides against *R. ferrugineus*. The current work seeks to investigate the growth kinetics of *O. basilicum* cell suspensions in order to develop and produce secondary metabolites. The phenolic composition of the components generated from *O. basilicum* culture was determined using LC-MS (total phenols, flavonoids, and (poly) phenolic acids). The insecticidal activity of *R. ferrugineus* larvae and adults is investigated in vivo and in vitro for contact insecticide and antifeedant action, as well as inhibition of the red palm weevil’s serine, cysteine, and metalloproteinases. In silico ADMET property evaluation (absorption, distribution, metabolism, excretion, and toxicity) as well as molecular docking occurred. The findings are likely to culminate in the development of an eco-friendly natural bio-insecticide to combat this pest.

## 2. Results

### 2.1. Initiation of Callus and Cell Suspension

**Figure 1:**
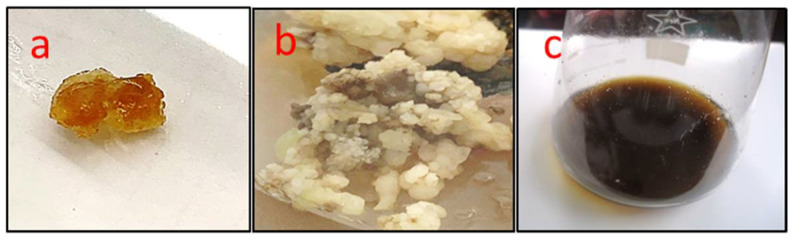
*O. basilicum* callus and cell suspension in MS and LS medium containing kinetin (0.5 mg/L), NAA (0.1 mg/L), 2,4-D (0.1 mg/L), and IBA (1 mg/L) and 3% (*w*/*v*) sucrose; (**a**) callus induction after seven days, (**b**) callus induction after 25 days, and (**c**) cell suspension after 40 days via somatic embryogenesis. NAA: 1-Naphthaleneacetic acid; 2,4-D: 2,4-Dichlorophenoxyacetic acid; and IBA: Indole butyric acid.

The embryogenic calli were used to start cell suspension culture. It was found that the callus was proliferated and was, subsequently, transferred to the cell suspension medium at the age of five–six weeks. It is clear that the LS liquid media came out more often and had more somatic embryos than the solid media used for that purpose. Embryogenic calli were transferred to a cell suspension culture for proliferation for five–six weeks, where the rate and quantity of somatic embryos produced and initiated were more than what was obtained from solid media. Additionally, it was observed that MS and calli undergo an oxidative process, resulting in the appearance of a brown color, and that when macronutrients are used in their entirety, a fraction of the physically somatic embryos created grow (Figure 1). The liquid media and calli were brown at the start of initiation, and the color gradually darkened, showing that the formation of phenolic components in the media and the initiation process are time-dependent processes (Figure 1).

### 2.2. Chemical Content and Chemical Composition Analyses of O. basilicum Extract

The total phenolic content in infected callus and cell suspension of *O. basilicum* was 14.85 and 32.51 mg/g dried weight, respectively, which increased significantly and continued to rise until the end of the 40-day test period with inoculation of *V. dahlia* (Table 1). The results presented in callus and cell suspension without infection were 9.78 and 19.23 mg/g dried weight, respectively. Overall, the total phenolic content of *O. basilicum* differs significantly (*p* ≤ 0.0005) with and without infection by *V. dahliae* (Table 1). The results in (Table 1) demonstrate that a higher value of total flavonoids was found in the cell suspension infected with *V. dahlia* (3.97 mg/g DW). On the contrary, the uninfected callus had a lower value of 0.95 mg/g DW. The lowest estimated values of the secondary metabolites, whether in suspension or callus, were found in total condensed tannin values, where the most significant value was 0.74 mg/g DW for the infected cell suspension of *O. basilicum*. In contrast, in *O. basilicum,* the lower values were obtained in the suspended state (0.24 mg/g DW).

### 2.3. Polyphenolic Acids and Flavonoids Compounds in O. basilicum Cell Suspension Extracts Using UPLC–I Class Coupled with Xevo TQD MS

The concentrations of polyphenolic acids and flavonoids in *O. basilicum* cell suspension extracts identify 27 compounds using molecular weight, retention time, and fragmentation profile according to the literature and software’s data bank; this approach allowed for (Table 2). The detected compounds were identified using the negative scan mode of the mass spectrometer; the scan took 45 min, and the results for the extract are summarized. More specifically, the most abundant peak in extracts was rosmarinic acid, a polyphenol in cell suspension of *O. basilicum* (14.2 mg/g DW) with a retention time of 11.60 min at *m/z* 359.08 (M-H), *m/z* 117, 135, 161, 179, and 197 were used to identify the items (rosmarinic acid). Nepetoidin (A and B) was presented in an extract with 6.84 and 5.72 mg/g DW. Nepetoidin A and B had a retention time of 25.53 and 25.67 min, respectively, at *m/z* 314.29 (M-H). The molecular ions were identified at *m/z* 133, 161, 313, and 335 for nepetoidin A and 133, 161, 269, 313, and 335 for nepetoidin B. Basil cell suspension extract contained ursolic acid (4.91 mg/g DW), a polyphenol. It retained 26.06 min at *m/z* 456.7 (M-H), and the resultant ions had *m/z* 455, 456, 523, 524, and 591. Salvigenin (5-Hydroxy-6,7,4′-trimethoxyflavone was observed extracted with 2.51 mg/g DW at *m/z* 327.215 (M-H), a retention time of 18.29 min, and resultant ions at *m/z* 116.9, 205, 215, 277, and 311. Quercetin-3-O-rutinoside (Rutin), a flavonoid, was observed extracted with 2.34 mg/g DW at *m/z* 611.16 (M-H), a retention time of 11.53 min, and resultant ions at *m/z* 465, 449, and 303. Rosmarinic acid glucoside A and B were present in the cell suspension extract of basil with 1.84 and 1.45 mg/g DW, respectively. It had a retention time of 21.37 and 25.07 min at *m/z* 521.12 (M-H), and the resultant ions were identified at *m/z* 135, 161, 179, 197, and 359 for rosmarinic acid glucoside A as well as 135, 161, 179, 197, 323, and 359 for rosmarinic acid glucoside. Nepetoidin glucoside was present in the extract with 1.23 mg/g DW and a retention time of 27.99 min at *m/z* 475.12 (M-H). The presence of molecular ions was discovered at *m/z* 151, 161, 313, 323, and 475. Acidic compounds such as chicoric acid (dicaffeoyl-tartaric acid) and isocitric acid were significantly presented in the cell suspension extract at *m/z* 473, 311, 293, 179, and 149 at a retention time of 11.18 min of chicoric acid. Isocitric acid was present with a retention time of 2.54 min, *m/z* 191.01 (M-H), with resultant ions at *m/z* 111, 129, and 173 (Figure S1).

Additionally, other components were discovered in the extracts as naringenin 7-0-glucoside, apigenin 7-O-glucoside, cyanidin 3-O-rutinoside (cyaninoside), cyanidin 3,3’-diglucoside, and apigenin 7-O-glucoside with *m/z* 434.4, 432.4, 595.17, 611.16, and 432.4 (M-H), respectively. The salvianolic acid chemicals found in extracts included the following: salvianolic acid F (*m/z* 313.07 (M-H)), salvianolic acid B (*m/z* 717.15 (M-H)), salvianolic acid A (*m/z* 493.11 (M-H)), salvianolic acid E (*m/z* 717.15 (M-H)), salvianolic acid K (*m/z* 555.11 (M-H)), and salvianolic acid H/I (*m/z* 537.10 (M-H)). Other acids found were lithospermic acid, fertaric acid, caffeic acid, caftaric acid (caffeoyl-tartaric acid), caffeic acid derivative (3TMS), and tartaric acid, with *m/z* of 537.10, 325.06, 179.03, 311.04, 359.70, and 149.0076, respectively [24,37,38,39,40,41,42,43,44,45,46,47,48,49,50,51].

**Figure 2:**
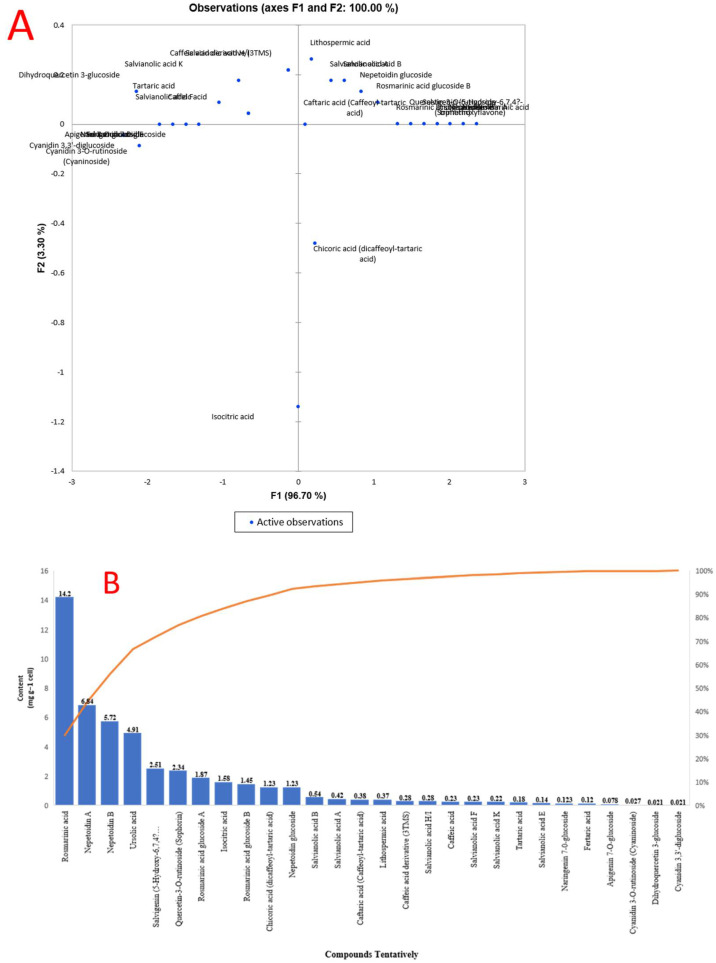
Analysis of the metabolite profiles of *O. basilicum* cell suspension extracts (**A**) principal component analysis (PCA) and (**B**) compounds tentatively and content of *O. basilicum* cell suspension extracts using LC-MS Chromatogram.

Principal component analysis (PCA) was performed to validate the differences between samples and the contribution of the metabolites to clustering. Figure 2 demonstrates no difference between *O. basilicum* extracts. The first component (PC1) accounts for 96.70% of variance, whereas the second component (PC2) accounts for 3.30%. The secondary metabolites that contributed the most to the cluster of *O. basilicum* extracts were acids, contain a high concentration of rosmarinic acid, nepetoidin (A and B), ursolic acid, salvigenin, quercetin-3-O-rutinoside, rosmarinic acid glucoside A and B, nepetoidin glucoside, and acidic compounds such as isocitric acid and chicoric acid (Figure 2).

### 2.4. O. basilicum Extract and Pure Compounds Activity against Adults and Larvae of R. ferrugineus

Table 3 shows the extract’s efficiency against adult (*R. ferrugineus*). The *O. basilicum* extract was shown to be active against adults with an LC_50_ of 1238 µg/mL and 95% confidence limits of 1038–1389. The LC_50_ values of chicoric acid, ursolic acid, salvigenin, quercetin-3-O-rutinoside, rosmarinyl glucoside, and nepetoidin B had the highest insecticidal activity with LC_50_ 1132, 1167, 1189, 1214, 1275, and 1317 µg/mL, and 95% confidence limits of 1004–1198, 1038–1204, 1049–1219, 1089–1234, 1147–1315, and 1268–1346, respectively. Adults’ moderate, low activity was presented in rosmarinic acid and isocitric acid with an LC_50_ of 1495 and 1826 µg/mL, respectively. The topical application showed that the LD_50_ value of *O. basilicum* extracts (µg/larva) was 13.7. Chicoric acid, salvigenin, nepetoidin B, and rosmarinic acid showed the highest insecticidal activity with LD_50_ values of 10.23, 11.4, 11.9, and 12.4 µg/larva, respectively. Ursolic acid, quercetin-3-O-rutinoside, and rosmarinyl glucoside all demonstrated moderate activity on the larva, with LD50 values of 15.2, 16.9, and 17.6 g/larva, respectively. Finally, isocitric acid showed low activity with an LD_50_ of 23.9 µg/larva.

### 2.5. Evaluation Specific Activity of O. basilicum Extract and Pure Compounds on Serine, Cysteine, and Metalloproteinase (In Vitro)

**Figure 3:**
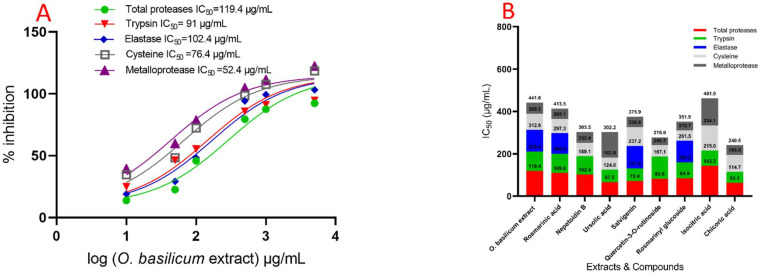
IC_50_ of *O. basilicum* extract from cell suspensions and rosmarinic acid, nepetoidin B, ursolic acid, salvigenin, quercetin-3-O-rutinoside, rosmarinyl glucoside, isocitric acid, and chicoric acid against total proteases, trypsin-like serine proteinase, chymotrypsin-like serine proteinase, elastase, cysteine protease, and metalloprotease (in vitro) of *R. ferrugineus* midgut, (**A**) activity, with IC_50_ of *O. basilicum* extract against proteases enzymes (in vitro), and (**B**) IC_50_ of *O. basilicum* extract and compounds against proteases’ enzymes (in vitro), according to one-way analysis of variance on the enzyme activity data (ANOVA).

**Figure 4:**
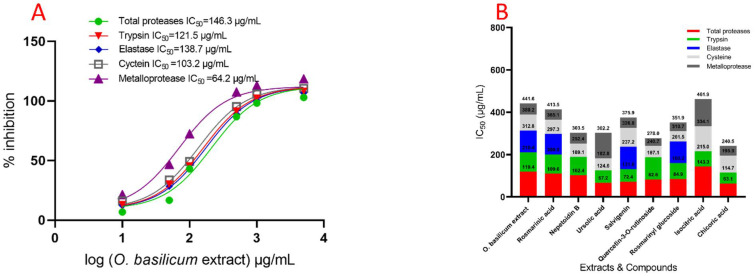
IC_50_ of *O. basilicum* extract from cell suspensions and rosmarinic acid, nepetoidin B, ursolic acid, salvigenin, quercetin-3-O-rutinoside, rosmarinyl glucoside, isocitric acid, and chicoric acid against total proteases, trypsin-like serine proteinase, chymotrypsin-like serine proteinase, elastase, cysteine protease, and metalloprotease (in vivo) of *R. ferrugineus* midgut. (**A**) activity, with IC_50_ of *O. basilicum* extract against proteases enzymes (in vivo), and (**B**) IC_50_ of *O. basilicum* extract and compounds aganist proteases enzymes (in vivo), according to one-way analysis of variance on the enzyme activity data (ANOVA).

The data demonstrated that serial doses of *O. basilicum* extract had an effect on IC_50_ values, as compared with untreated proteases from the midgut of larvae. Figure 3 demonstrated unequivocally that the IC_50_ of the midgut increased with concentrations. The IC_50_ value for *O. basilicum* extract was 119.4 µg/mL. The IC_50_ value for *O. basilicum* extract was 119.4 µg/mL. Figure 4 depicts the relative activity and inhibition of trypsin, chymotrypsin, and elastase-like proteinases in the fourth instar midgut homogenate. Serine proteinases have comparable specific activities in total homogenate preparations and are expressed as the number of OD/mg protein min. All IC_50_ values had a significant influence of *O. basilicum* extracts. The IC_50_ values in inhibition by *O. basilicum* extract is substantially greater for trypsin-like serine proteinases and elastase than for chymotrypsin-like serine proteinase in midgut homogenate preparations. The IC_50_ value for *O. basilicum* extract is shown in Figure 4, which demonstrates that the inhibition varies greatly amongst homogenates of the midgut. In OD/mg protein min from fourth instar midgut preparation, trypsin-like serine and elastase proteinase activities were 4.10 and 1.24, respectively. Moreover, *O. basilicum* was the active extract, with an IC_50_ of 91 and 102.4 µg/mL, respectively. These extracts have a potent inhibitory effect on trypsin-like serine proteinases isolated from the fourth midgut. Both cysteine and metalloprotease are a strong inhibitory response to *O. basilicum* extract; the IC_50_ values are shown in Figure 4, which demonstrates that the extract exhibits inhibitory activity at IC_50_ 100 µg/mL with values of 76.4 and 52.4 µg/mL, respectively. Chymotrypsin-like serine proteinases have a distinct pattern of activity than serine activity; the IC_50_ values for chymotrypsin are shown in Figure 4, which demonstrates that the extract did not have an inhibition effect against chymotrypsin when the IC_50_ value is more than 5000 µg/mL.

Figure 3 depicts the activity of compounds on total protease activity (in vitro) isolated from the midgut of *R. ferrugineus* fourth instar larvae. Different dosages had a substantial significant effect on the IC_50_ rates for all investigated compounds when compared to untreated larvae. The compounds’ effect on total protease activity (in vitro) of *R. ferrugineus* fourth instar larvae midgut is shown in Figure 4. Different IC_50_ rates for all investigated compounds had a substantial influence. Chicoric acid, ursolic acid, salvigenin, quercetin-3-O-rutinoside, rosmarinyl glucoside, nepetoidin B, rosmarinic acid, and isocitric acid were presented activity against total proteases, with IC_50_ values of 63.1, 67.2, 72.4, 82.6, 84.9, 102.4, 109.6, and 143.3 µg/mL, respectively. Chicoric acid, ursolic acid, salvigenin, rosmarinyl glucoside, nepetoidin B, rosmarinic acid, and quercetin-3-O-rutinoside demonstrated a more significant effect against trypsin, with IC_50_ values of 51.6, 57.4, 59.2, 75.3, 86.7, 91.3, and 104.5 µg/mL, respectively. Rosmarinic acid, rosmarinyl glucoside, and salvigenin inhibited elastase activity, with IC_50_ values of 96.4, 101.3, and 105.6 µg/mL, respectively, and the remind tested compounds did not have an inhibition effect with IC_50_ > 5000 µg/mL. Figure 4 illustrates the IC_50_ values for chymotrypsin, denoting that all compounds did not have an inhibition effect with IC_50_ > 5000 µg/mL. Metalloproteases have a specific and high inhibition response to quercetin-3-O-rutinoside, rosmarinyl glucoside, chicoric acid, rosmarinic acid, salvigenin, and nepetoidin B. The IC_50_ values showing that compounds have the highest inhibition where IC_50_ ˂ 100 µg/mL, with values of 37.3, 41.2, 44.6, 48.4, 49.1, and 51.1 µg/mL, respectively (Figure 3). Furthermore, ursolic acid and isocitric acid showed moderated activity against metalloproteases, with IC_50_ values of 119.4 and 127.8 µg/mL, respectively. Moreover, cysteine demonstrated an inhibition response to rosmarinyl glucoside, quercetin-3-O-rutinoside, ursolic acid, nepetoidin B, rosmarinic acid, chicoric acid, and salvigenin compounds, which presented the highest inhibition with values 49.2, 53.6, 58.2, 63.3, 67.8, 81.2, and 89.6 µg/mL, respectively (IC_50_ ˂ 100 µg/mL). Isocitric acid was lower in IC_50_ value with 119.1 µg/mL.

These were included in proteolysis activity evaluation, to determine the involvement of several intestinal proteases in the fourth larval instars when protease inhibitors were used. Purified proteases from the fourth larval instars of *R. ferrugineus’s* midgut were significantly reduced by inhibitors, as shown in Figure S2. Additionally, the inhibitors of trypsin-like serine proteinases TLCK and chymotrypsin-like serine proteinases TPCK, as well as the cysteine protease inhibitor iodoacetic acid, dramatically suppressed the midgut instar (Figure S2).

### 2.6. In Vivo Effect of Specific Protease Inhibitors and O. basilicum Extract on the Serine, Metalloprotease, and Cysteine Protease Activities from Fourth R. ferrugineus Instar Midgut Preparations

The IC_50_ value of the fourth instar larvae midgut of *R. ferrugineus* increased gradually with concentrations of *O. basilicum* extract. The *O. basilicum* extract showed a significant influence effect according to the IC_50_ value (Figure 4). The activity was 1.92 in OD/mg protein min from preparations of the fourth instar midgut. The inhibition by *O. basilicum* extract was demonstrated in the trypsin-like serine, elastase proteinases, metalloprotease, and cysteine protease from the fourth midgut preparation values, which were higher than in vitro values. The activity of *O. basilicum* extract against total proteases, trypsin-like serine proteinase, and elastase was shown by IC_50_ values of 146.3, 132.5, and 138.7 µg/mL, respectively.

**Figure 5:**
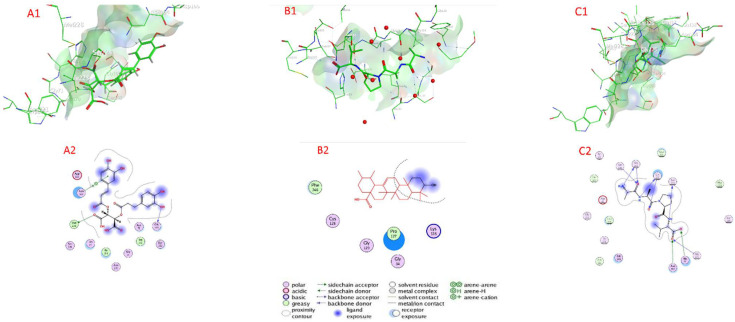
Docking view of (**A1**,**A2**) chicoric acid, (**B1**,**B2**) ursolic acid, and (**C1**,**C2**) salvigenin, in the binding sites of serine proteinase (PDB:2F7O); (**A1**,**B1**,**C1**) 3D complex structures (stereoview) and (**A2**,**B2**,**C2**) 2D interaction diagram structures.

The inhibition effect of cysteine and metalloprotease indicates that the extract has the highest inhibitory effect when IC_50_ ˂ 105 µg/mL, with values of 103.2 and 64.3 µg/mL, respectively (Figure 4). Data displayed in Figure 4 demonstrate the compound’s effect on total protease activity (in vivo). The chicoric acid, salvigenin, nepetoidin B, and rosmarinic acid present activity against total proteases, having IC_50_ values of 146.2, 164.8, 179.4, and 186.2 µg/mL, respectively. Ursolic acid, quercetin-3-O-rutinoside, rosmarinyl glucoside, and isocitric acid activity against total protease shows that compounds have the lowest inhibition when IC_50_ is 279.5, 286.4, 292.1, and 486.3 µg/mL. Chicoric acid, Nepetoidin B, and rosmarinic acid have a more substantial effect against trypsin, having IC_50_ values of 75.1, 102.7, and 174.6 µg/mL, respectively. In addition, ursolic acid, salvigenin, rosmarinyl glucoside, isocitric acid, and quercetin-3-O-rutinoside have a non-significant effect against trypsin, having IC_50_ values of 235.1, 241.2, 274.3, 314.8, and 325.5 µg/mL, respectively. The elastase activity indicates inhibition effects with rosmarinic acid, salvigenin, and rosmarinyl glucoside, having IC_50_ values of 119.7 and 138.2, 751.7 µg/mL, respectively, and remind tested compounds have the most non-significant inhibition where IC_50_ > 5000 µg/mL. Metalloproteases have an inhibition response with chicoric acid, rosmarinic acid, nepetoidin B, salvigenin, and isocitric acid; the IC_50_ values are presented in Figure 5, which indicates compounds have inhibition where IC_50_ is 74.2, 86.7, 95.8, 113.7, and 169.2 µg/mL, respectively. Furthermore, ursolic acid, quercetin-3-O-rutinoside, and rosmarinyl glucoside showed less activity against metalloproteases having IC_50_ 548.3, 612.4, and 643.5 µg/mL, respectively. Moreover, cysteine has shown an inhibition response to nepetoidin B, salvigenin, rosmarinic acid, chicoric acid, and isocitric acid (Figure 5), with IC_50_ ˂ 100 µg/mL and values of 109.5, 126.7, 131.5, 157.6, and 174.2 µg/mL, respectively. Moreover, ursolic acid, quercetin-3-O-rutinoside, and rosmarinyl glucoside demonstrated less activity against IC_50_ of 685.2, 746.6, 876.8, and 1163.2 µg/mL, respectively. Chymotrypsin-like serine proteinases did not present the same trend, as shown in the specific activity of serine proteinase, metalloprotease, and cysteine protease; the IC_50_ values of chymotrypsin are presented in Figure 5, indicating that extract did not have an inhibition effect in chymotrypsin when IC_50_ > 5000 µg/mL.

### 2.7. Docking of Compounds into Proteinase Enzymes

#### 2.7.1. Serine Proteinase Docking

Table 4 as well as Figure 5 and Figure S3 show the docking scores of the compounds with serine proteinase (PDB:2F7O). The docking analysis revealed that the investigated compounds had a high affinity for the serine protease’s active sites (target enzyme) and a low docking energy ranging from −4.4249 (isocitric acid) to −6.8202 (chicoric acid) kcal/mol for the target enzyme (Table 4). Chicoric acid, ursolic acid, salvigenin, nepetoidin A, and quercetin-3-O-rutinoside had higher binding affinity than compounds with the lower docking energy range, with values of −6.8202, −6.6226, −6.2654, −6.0523, and −6.0175 kcal/mol, respectively, followed by nepetoidin B, rosmarinic acid, and rosmarinic acid glucoside with docking energies of −5.8765, −5.7938, and −5.6609 kcal/mol, respectively. In contrast, with a docking energy of −4.4249 kcal/mol, isocitric acid has the lowest binding affinity for serine proteinase enzyme (Table 3). Chicoric acid connected to the active sites of serine proteinase via H– bonds with Met 228 (3.71 Å) Gly 103 (3.04 Å), and an H-pi bond with Asn 164 (3.81 Å), as well as van der Waals contacts with many amino acids including Asn 70, Ser 102, Try 215, Gly 71, Asn 221, Ile 223, His 72, Ser 224, and Asp 165. Ursolic acid connected to the active sites of serine proteinase via van der Waals contacts with amino acids Lys 128, Pro 127, Gly 34, Gly 129, Cys 126, and Phe 246. Salvigenin connected to the active sites of serine proteinase via H– bonds with Gly 105 (3.10 Å), Gly 137 (2.90 Å), Ser 135 (3.27 Å), Ser 140 (3.80 Å), Gly 105 (3.46 Å), Gly 137 (2.94 Å), Asn 164 (2.54 Å), Gly 225 (3.55 Å), His 72 (2.25 Å), and Asn 70 (3.27 Å), as well as van der Waals, was contacts with many amino acids including Gly 103, Ala 161, Thr 226, Gly 138, Asp 41, Ile 110, Tyr 107, Leu 99, Met 228, and Ser 227.

#### 2.7.2. Cysteine Protease Docking

**Figure 6:**
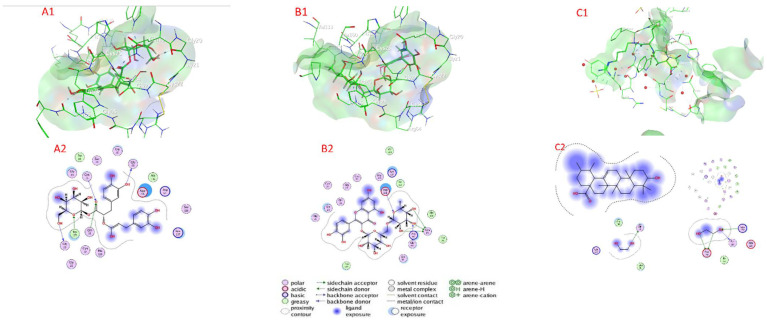
Docking view of (**A1**,**A2**) rosmarinic acid glucoside, (**B1**,**B2**) quercetin-3-O-rutinoside, and (**C1**,**C2**) ursolic acid in the binding sites of cysteine protease (PDB:3IOQ); (**A1**,**B1**,**C1**) 3D complex structures (stereoview), and (**A2**,**B2**,**C2**) 2D interaction diagram structures.

Table 4 as well as Figure 6 and Figure S4 show the docking scores of the compounds with cysteine protease (PDB:3IOQ). The docking analysis revealed that the investigated compounds had a high affinity for the cysteine protease’s active sites (target enzyme) and low docking energy ranging from −4.1368 (isocitric acid) to −6.5869 (rosmarinic acid glucoside) kcal/mol for the target enzyme (Table 4). Rosmarinic acid glucoside, quercetin-3-O-rutinoside, ursolic acid, and rosmarinic acid had higher binding affinity than compounds with a lower docking energy range, with values of −6.8202, −6.5665, −6.4766, and −6.4056 kcal/mol, respectively, followed by chicoric acid, nepetoidin B, salvigenin, and nepetoidin A with docking energies of −5.8215, −5.4078, −5.3786, and 5.2341 kcal/mol, respectively. In contrast, with a docking energy of −4.1368 kcal/mol, isocitric acid has the lowest binding affinity for cysteine protease enzyme (Table 3). Rosmarinic acid glucoside Chicoric acid connected to the active sites of cysteine protease via H– bonds with Gly 66 (2.85 Å), Gly 20 (3.04 Å), Gln 19 (3.21 Å), Cyc 25 (3.39 Å), and Trp 177 (2.89 Å), as well as van der Waals interactions with a large number of amino acids Gly 21, Cys 22, Gly 23, Ser 24, Tip 26, Gly 65, Ala 136, Ser 156, His 159, Arg 64, Lys 137, and Asp 158. Quercetin-3-O-rutinoside connected to the active sites of cysteine protease via H– bonds with Cyc 25 (3.50 Å), Cyc 25 (2.60 Å), Cyc 25 (3.66 Å), Asp 158 (3.36 Å), and Gly 66 (3.29 Å), as well as van der Waals contacts with amino acids (Gly 21, Gly 32, Gln 19, Cys 22, Gly 20, Cys 63, His 159, Lys 137, Ile 157, Val 133, Ala 160, Trp 26, Gly 65, and Trp 177). Ursolic acid connected to the active sites of cysteine protease via H– bonds with Gly 66 (3.64 Å), Cyc 25 (3.50 Å), Lys 137 (3.12 Å), Arg 64 (3.19 Å), Gln 19 (3.20 Å), Gly 20 (3.26 Å), Ser 156 (2.86 Å), Asp 158 (3.34 Å), Asp 158 (3.36 Å), and Gln 44 (3.03 Å), as well as van der Waals, with interactions with a large number of amino acids: Gly 23, Cys 22, Gly 65, Gln 19, Asp 158, Tyr 61, Gln 44, and Gly 66.

#### 2.7.3. Metalloprotease Docking

**Figure 7:**
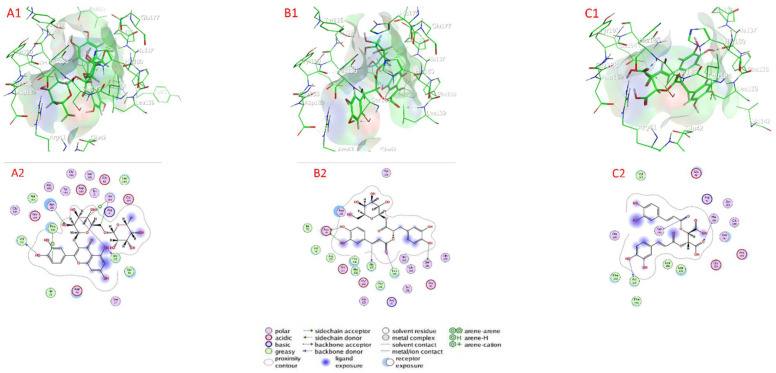
Docking view of (**A1**,**A2**) quercetin-3-O-rutinoside, (**B1**,**B2**) rosmarinic acid glucoside, and (**C1**,**C2**) chicoric acid in the binding sites of metalloprotease (PDB:1KAP); (**A1**,**B1**,**C1**) 3D complex structures (stereoview) and (**A2**,**B2**,**C2**) 2D interaction diagram structures.

Table 4 as well as Figure 7 and Figure S5 show the docking scores of the compounds with metalloprotease (PDB:1KAP). The docking analysis revealed that the investigated compounds had a high affinity for the metalloprotease’s active sites (target enzyme) and low docking energy ranged from −4.3629 (isocitric acid) to −8.1833 (rosmarinic acid glucoside) kcal/mol for the target enzyme (Table 4). Quercetin-3-O-rutinoside, rosmarinic acid glucoside, chicoric acid, rosmarinic acid, salvigenin, and nepetoidin B had higher binding affinity than compounds with the lower docking energy range, with values of −8.1833, −7.7259, −6.2654, −6.3303, −6.3212, −6.2783, and −6.0265 kcal/mol, followed by nepetoidin A, and ursolic acid with docking energies of −5.9404 and −4.9541 kcal/mol, respectively. In contrast, with a docking energy of −4.3629 kcal/mol, isocitric acid has the lowest binding affinity for metalloprotease enzyme (Table 3). Quercetin-3-O-rutinoside connected to the active sites of metalloprotease via H– bonds with Ala 137 (2.84 Å), Val 131 (3.02 Å), Asn 191 (2.95 Å), and an H-pi bond with His 180 (3.52 Å), Phe 136 (4.52 Å), and His 180 (4.21 Å), as well as van der Waals contacts with many amino acids (Asp 78, Ile 79, Ser 77, Glu 177, Arg 41, Leu 139, Tyr 75, Glu 42, Asp 189, Ser 185, Gly 188, Try 190, His 186, Ala 192, Glu 194, and Gly 193. Rosmarinic acid glucoside connected to the active sites of metalloprotease via H– bonds with Ala 137 (3.27 Å), Asp 78 (3.10 Å), Asp 78 (2.92 Å), Ser 185 (3.01 Å), Leu 139 (3.33 Å), as well as van der Waals contacts with amino acid (Ile 79, Val 76, Ala 135, Glu 177, Phe 136, Ala 192, Asp 189, His 176, Phe 138, Arg 41, Tyr 190, His 180, Gly 188, Glu 42, and His 188), Chicoric acid connected to the active sites of metalloprotease via H– bonds with Ala 137 (2.95 Å), His 186 (2.88 Å), and Ser 185 (3.28 Å), as well as van der Waals, with contacts with amino acids Glu 42, Arg 41, Tyr 190, Gly 188, Asn 188, Asp 189, Glu 194, Leu 139, Leu 184, Phe 136, Phe 138, His 180, and Val 142.

### 2.8. ADMET Analysis

Table S1 summarizes the examination of several descriptors, such as HBA, HBD, LogP, LogS, PPB, BBB, CYP450, and H-HT of all compounds, which are displayed in Table S1. Chicoric acid, salvigenin, nepetoidin A, nepetoidin B, and rosmarinic acid with LogP values 1.228–3.191 are present in Table S1. On the other side, quercetin-3-O-rutinoside, rosmarinic acid glucoside, and isocitric acid have LogP values < 0, and all LogS values are more than 10 μg/mL. The LogS, and LogP values of ursolic acid equal 0.187 μg/mL and 7.09, respectively. (Table S1). In addition, nepetoidin A, nepetoidin B, ursolic acid, salvigenin, and isocitric acid demonstrated elevated polar surface area (PSA), indicating poor cell membrane penetration ability with PSA values 107.22, 107.22, 57.53, 78.13, and 132.13, respectively. On the contrary, rosmarinic acid, rosmarinic acid glucoside, quercetin-3-O-rutinoside, and chicoric acid have a polar surface area (PSA), indicating good cell membrane penetration ability with PSA values 144.52, 223.67, 269.43, and 208.12, respectively. Furthermore, CYP450 has a negative correlation with nepetoidin A, nepetoidin B, ursolic acid, chicoric acid, and isocitric acid, and has a positive correlation with rosmarinic acid (+), salvigenin (++), and rosmarinic acid glucoside (+). PPB for all compounds demonstrated elevated positive. Additionally, CYP450 and H-HT have positive values with all compounds. The hepatotoxicity has a positive relationship with rosmarinic acid, nepetoidin A, nepetoidin B, and salvigenin, whereas H-HT of nepetoidin A, nepetoidin B, ursolic acid, chicoric acid, and isocitric acid was negative.

## 3. Discussion

The current study examined the secondary metabolites from cell suspensions extract of *O. basilicum*. Chicoric acid, ursolic acid, salvigenin, quercetin-3-O-rutinoside, rosmarinyl glucoside, and nepetoidin B possess a high degree of insecticidal action against adults of *R. ferrugineus*. Chicoric acid, salvigenin, nepetoidin B, and rosmarinic acid also demonstrated the highest topical application activity against larva. The study found that *O. basilicum* extracts were efficacious against total proteases, present specific activity against trypsin-like serine proteases and elastases (in vitro and in vivo experiments). Secondary metabolite synthesis, including polyphenolic acids and flavonoids compounds, rose fast in *O. basilicum* cell suspensions, particularly under inoculation with *V. dahliae*. The *O. basilicum* extract was shown to be efficacious and effective, not only enhancing deterrence but also reducing feeding in *R. ferrugineus* larvae. However, its antifeedant action is most evident in adults.

Furthermore, our results indicated that under ideal settings, the strongest rates of antifeedant activity were found. Significant insecticidal activity was observed in the extract, which might be related to the extract’s variety of bioactive metabolites. *O. basilicum* polyphenolic acids and flavonoids from cell suspension grew gradually throughout the first 25 days, then rapidly increased and continued to rise fast over the last 15 days of the testing session. Different dosages had a substantial influence for compounds examined with IC_50_ rate. The impact of compounds against protease activity isolated in vitro from the midgut of *R. ferrugineus* fourth instar larvae explains the action of *O. basilicum*.

The LC_50_ value was 1238 µg/mL for *O. basilicum* extract against adults. The LC_50_ values of chicoric acid, ursolic acid, salvigenin, quercetin-3-O-rutinoside, rosmarinyl glucoside, and nepetoidin B had the highest insecticidal activity, with LC_50_ 1132, 1167, 1189, 1214, 1275, and 1317 µg/mL, respectively. The topical application showed that the LD_50_ value (µg/larva) was 13.7 for *O. basilicum* extracts. Chicoric acid, salvigenin, nepetoidin B, and rosmarinic acid demonstrated the highest insecticidal activity, with LD_50_ values of 10.23, 11.4, 11.9, and 12.4 µg/larva, respectively. Isocitric acid was present with low activity with LD_50_ 23.9 µg/larva.

The active extract of *Ocimum basilicum* showed inhibitory activity against total protease, trypsin-like serine proteinases, elastase, cysteine, and metalloprotease activity with IC_50_ values of 119.4, 91, 102.4, 76.4 and 52.4 µg/mL, respectively. However, the activity of *O. basilicum* extracts against proteases enzymes demonstrated a correlation with nepetoidin B (5.72%), quercetin-3-O-rutinoside (2.34%), chicoric acid (1.23%), rosmarinic acid (14.2%), rosmarinyl glucoside (3.32%), and salvigenin (2.51%) contents. Chicoric acid, ursolic acid, salvigenin, quercetin-3-O-rutinoside, rosmarinyl glucoside, nepetoidin B, rosmarinic acid, and isocitric acid presenting inhibition effects against total proteases, with IC_50_ values of 63.1, 67.2, 72.4, 82.6, 84.9, 102.4, 109.6, and 143.3 µg/mL, respectively. The *Ocimum basilicum* extract activity against trypsin is related to chicoric acid, ursolic acid, salvigenin, rosmarinyl glucoside, nepetoidin B, rosmarinic acid, and quercetin-3-O-rutinoside, with IC_50_ values of 51.6, 57.4, 59.2, 75.3, 86.7, 91.3, and 104.5 µg/mL (1.23%, 4.91%, 2.51%, 3.32%, 5.72%, 14.2%, and 2.31%), respectively. Quercetin-3-O-rutinoside, rosmarinyl glucoside, chicoric acid, rosmarinic acid, salvigenin, and nepetoidin B have the highest responsibility of inhibition effect against metalloproteases with IC_50_ ˂ 100 µg/mL, and values of 37.3, 41.2, 44.6, 48.4, 49.1 and 51.1 µg/mL, respectively. On the contrary, rosmarinyl glucoside, quercetin-3-O-rutinoside, ursolic acid, nepetoidin B, rosmarinic acid, chicoric acid, and salvigenin presented specific inhibitory effects against cysteine where IC_50_ < 100 µg/mL, with values of 49.2, 53.6, 58.2, 63.3, 67.8, 81.2, and 89.6 µg/mL, respectively. The inhibitory activities of rosmarinic acid, rosmarinyl glucoside, and salvigenin against elastase activity are demonstrated by IC_50_ values of 96.4, 101.3, and 105.6 µg/mL, respectively. These findings are consistent with the fact that compounds have an effect on total protease activity (in vitro, in vivo), such as nepetoidin B, quercetin-3-O-rutinoside, chicoric acid, rosmarinic acid, rosmarinyl glucoside, and salvigenin, which have activity against total proteases. It can be concluded that nepetoidin B, quercetin-3-O-rutinoside, chicoric acid, rosmarinic acid, rosmarinyl glucoside, and salvigenin were presented to effect insecticidal activity against proteases enzymes of *R. ferrugineus*.

Moreover, since these extracts are antifeedant chemicals, their targets and method of action in insects remain unclear. The research suggests that processes may include disturbance of feeding physiology, chronic toxicity, or repellency associated with the insecticidal activity [52]. The most significant secondary metabolites in *O. basilicum* extracts were flavonoids and polyphenolic acids, which were the major steps in the production pathway for flavonoid and polyphenolic precursors [53,54].

Notably, these secondary molecules are recognized as significant constituents after their formation on cells as a vital protection from pathogens [55,56]. PGR is required for the regulation of plant development and differentiation, which could manage the anabolism destruction of the phenolic contents [57,58]. As a result, novel techniques such as the green biosynthesis of the bioactive secondary metabolites are in great demand now [59,60,61]. Additionally, cell suspension culture is a more effective and quick approach for enhancing bioactive chemical synthesis than callus culture, owing to its rapid responsiveness, cell division, and simplicity of application [1,60,61]. Total proteolytic activity was evaluated in the midgut juice of *R. ferrugineus* larval instars using azocasein as the substrate with a slightly alkaline mixture (pH 8.0), which is physiologically similar to the insect midgut pH [1], and DTT activator was used [1,62]. The variations in the secondary metabolites of *O. basilicum* extracts toward protease inhibition in vitro and in vivo assays (Figure 3 and Figure 4) established the critical role proteinases in their mode of action [63]. The findings indicate that toxicity might exist as a consequence of nepetoidin B (5.72%), quercetin-3-O-rutinoside (2.34%), chicoric acid (1.23%), rosmarinic acid (14.2%), rosmarinic acid glucoside (3.32%), and salvigenin (2.51%) in extract *O. basilicum* and may attest to the critical nature of proteinases.

The inhibition of trypsin-like serine, elastase proteinases, metalloprotease, and cysteine protease by *O. basilicum* extract was clearly demonstrated in the fourth midgut preparation values, which were lower than the values in vitro. Many values in vivo have the same trend and are lower than the values in vitro. The in vivo assay of ursolic acid, quercetin-3-O-rutinoside, rosmarinyl glucoside, and isocitric acid activity against total protease demonstrated that compounds inhibit total protease the least in vitro. According to ADMET screening analysis, they are extremely hydrophobic, allowing them to permeate biological membranes according to the chicoric acid, salvigenin, nepetoidin A, nepetoidin B, and rosmarinic acid.

Further, Quercetin-3-O-rutinoside, rosmarinyl glucoside, and isocitric acid have poor lipid bilayer permeability according to LogP values (−1.687, −0.98 and 01.811, respectively). Compound ursolic acid has poor aqueous solubility (LogP 7.09), and low solubility (−6.387 log mol/L (0.187 μg/mL)). Based on these findings, the Lipinski rules indicate that chicoric acid, salvigenin, nepetoidin, nepetoidin B, and rosmarinic acid possess a high theoretical bioavailability in the oral. The effectiveness of compounds (chicoric acid, salvigenin, nepetoidin A, nepetoidin B, and rosmarinic acid) as a bio-insecticide is measured not only by its potential to attack the target insect but also by its absorption, distribution, metabolism, excretion, and toxicity risk (ADMET) profile. In terms of metabolism, nepetoidin A, nepetoidin B, ursolic acid, chicoric acid, and isocitric acid have no inhibition against CYP450 enzymes, indicating good metabolic stability against CYP450 enzymes, whereas rosmarinic acid, salvigenin, and rosmarinic acid glucoside have inhibition against CYP450 enzymes. However, the hepatotoxicity prediction suggests that the compounds rosmarinic acid, nepetoidin A, nepetoidin B, and salvigenin may be toxic to liver cells.

Docking analysis demonstrated that all of the examined ligands form hydrogen bonds between amino acids and target active pockets of serine proteinases, elastase, and cysteine protease enzymes [64]. Only metalloprotease exhibited ionic and metallic bonds with H-bonding with amino acids. The highest docking compound’s binding (chicoric acid, ΔG = −6.8202) to serine proteinase is exhibited. The binding confirmation of quercetin-3-O-rutinoside has the highest docking (ΔG = −6.5869) with cysteine protease. Rosmarinic acid glucoside (ΔG = −8.1833) also showed the highest docking with metalloprotease. ADMET assessment of the compounds under investigation revealed that chicoric acid, salvigenin, nepetoidin A, nepetoidin B, and rosmarinic acid met ADMET descriptors at their optimum level, as determined by in silico studies [45]. The Molecular Docking is explained as a technique for predicting binding affinities and interactions between molecules by studying their location or orientation on possible targets. The docking of the compounds on serine proteinases, elastase, metalloprotease, and cysteine protease revealed a variety of interactions, including H– bonds as well as H-pi hydrophobic and van der Waals interactions. These previous bond interactions assisted in the understanding of a variety of chemicals’ biological functions in a variety of domains, including medications and insecticides [45].

The extract of *O. basilicum* show activity according to the LC_50_ values against *R. ferrugineus*. The *O. basilicum* extract demonstrated obvious antifeedant and insecticidal action, and, clearly, inhibited proteinases isolated from the fourth midgut preparation. These results give insight into components that might be utilized to create biochemical markers that indicate the resistance of particular plants to insect infestation.

## 4. Materials and Methods

### 4.1. Chemicals and Reagents

The chemicals were provided by Merck Chemical Co. (St. Louis, MO, USA); hypochlorite solution, Hydrochloric acid, gallic acid, cyanidin, quercetin, and solvents were obtained from Sigma-Aldrich Chemical Co. (St. Louis, MO, USA), whereas Sigma-Aldrich Chemical Co. (St. Louis, MO, USA) provided reagents, media, and chemicals for biochemical studies. Rosmarinic acid, ursolic acid, quercetin-3-O-rutinoside (Rutin), isocitric acid, and chicoric acid were obtained from Merck Chemical Co. (St. Louis, MO, USA). Rosmarinyl glucoside was obtained from Aobious Inc., 9 Blackburn Drive, Gloucester, MA 01930 (USA). Nepetoidin B, salvigenin, and rosmarinyl glucoside were obtained from BioCrick Biotech, 88 Keyuan Road, Hi-Tech Zone, Chengdu, Sichuan 610042, PRC.

### 4.2. Media

Several types of media were used. Murashige and Skoog (MS) as solid medium and Linsmaier and Skoog (LS) as liquid medium (including 0.1 mg thiamine HCl and 100 mg myo-inositol) were obtained from Sigma-Aldrich Chemical Co. (St. Louis, MO, USA). Various media were made then autoclaved for 20 min (121 °C). The Microbiology Laboratory, College of Agricultural and Food Sciences, King Faisal University, Al-Ahsa, Saudi Arabia contributed the *V. dahliae* strain. Pronadisa, Madrid, Spain, provided the potato dextrose agar (PDA), tryptone, and yeast extract, while Sigma Chemical Co., St. Louis, MO, provided the agar (USA).

### 4.3. Plants

During the months of February and March, *O. basilicum* seeds were obtained from commercial nurseries in Al-Ahsa, Saudi Arabia. Sterilized seeds were put in MS medium, including agar (0.6%), with pH 5.7 with 3% sucrose (3%, *w*/*w*), and incubated in a climate room (26 ± 4 °C, 16 h light) for 7–8 weeks (seedling 17–20 cm) at King Faisal University’s Research and Training Station in Al-Ahsa, Saudi Arabia.

### 4.4. O. basilicum Calluses Initiation Employing Various PGRs in Conjunction and V. dahliae as a Biotic Elicitor

The seeds of *O. basilicum* were germinated in a Petri dish lined after being sterilized, delinted using paper for sterile blotting at a temperature of 28 ± 2 °C and a light intensity of 30 Einsteins/(m^2^·s). The explants of *O. basilicum* (epicotyls, hypocotyls, and cotyledonary, 4–5 mm in length) were put in an MS medium including kinetin (0.5 mg/L), NAA (1-Naphthaleneacetic acid) (0.1 mg/L), 2,4-D (2,4-Dichlorophenoxyacetic acid) (0.1 mg/L), IBA (Indole butyric acid) (1 mg/L), and sucrose (3%, *w*/*v*) according to Darrag et al., per Muhammed Akif Açıkgöz with modifications [1,32]. Every treatment (except control without PGRs) was maintained for eight weeks in a climate chamber (26  ±  4  °C, 16 h light) with three weekly subcultures. According to Darrag et al. [1], *V. dahliae* was utilized as an initiator (biotic elicitor) to explore callus growth enhancement. Vacuum filtration was used to extract callus from individual cultures 72 h after infection, and for 40 days Calli was visually examined every five days. *V. dahliae* was cultured in PDA (22 °C) and subculture every 5–6 weeks. Conidia were cultivated for 10 days at 22 °C in a potato dextrose (PD) medium with 240 rpm by rotary shaker. Conidia were extracted using centrifugation and rinsed three times at pH 6.5 with 0.1 M K_2_HPO_4_-KH_2_PO_4_. Conidia concentration was determined using a hemocytometer under a microscope. Finally, 25 µL of conidial suspension [(2–5) × 10^7^ conidia/mL] or sterile distilled water (control) was injected into 8 mL of fresh solid medium of MS.

### 4.5. O. basilicum Cell Suspension Initiation

According to Darrag et al. with modifications [1], callus was started to initiate and be detected throughout a 5–6-week period utilizing LS medium. The medium was passed through a variety of mesh sizes of screens. Liquid LS culture (200 mL) was filtered and added with either 25 mL conidial suspension [(2–5) × 10^7^ conidia/mL], then transferred to 30 flasks (Erlenmeyer, 500 mL), and adjusted to a final volume of 250 mL using liquid medium. After 72 h, cultures were collected and tested for protein content. Suspension media was established in conical flasks (250 mL) using LS medium (100 mL) without agar, maintained for six weeks at the conditions 30 ± 4 °C, with 16 h of light with a quick shaker speed 110 rpm, every two weeks, while the subculture was kept in a climate chamber.

### 4.6. Total Phenolic Content (TPC) Determination

The total phenolic components from samples (callus and cell suspension) were extracted using methanol by shaking for 12 h and followed by drying using anhydrous Na_2_SO_4_. Next was the addition of 790 µL distilled water to 10 µL sample solution and the 50 µL Folin–Ciocalteu reagent (50 L), according to the Folin–Ciocalteu technique [65]. The mixture was homogenized, and 150 µL of Na_2_CO_3_ solution (20%, *w*/*v*) was applied (1 min), vortexed again, and incubated at room temperature (120 min in dark). The total phenolic content was evaluated using spectroscopy at 750 nm and estimated using the standard curve of 1–100 mg gallic acid per 100 g·Dw.

### 4.7. Total Flavonoids Contents (TF)

The content in methanol extract was evaluated by extracting (500 µL) in mixture of extract and 5% (*w*/*v*) of sodium nitrite (500 µL), and, then, adding aluminum chloride (300 µL, 10% (*w*/*v*)) [40] from the cell suspension and callus samples [66]. The assay mixture is incubated for 5 min at ambient temperature, then sodium hydroxide (1 mL, 1 M) was added to bring the process to a halt. Total flavonoid content was evaluated by spectroscopy at 510 nm and calculated using standard curve of quercetin with a serial concentration of quercetin (1–100 mg quercetin/100 g·DW).

### 4.8. Determination of Total Condensed Tannins (TCT)

Total condensed tannins in methanol extract were determined according to Hagerman [67] from the cell suspension and callus samples. Ferric ammonium sulfate or ferric reagent (3000 µL, 2%, *w/v* in 2 M HCl) was added to extract sample (500 µL), butanol–HCl reagent (100 µL, 95:5) and mixed. The sample was kept at 90 °C for 15 min, then the total condensed tannins content was determined by spectroscopy at 550 nm and calculated using a serial concentration of cyanidin (0.1–10 mg of cyanidin/100 g·DW).

### 4.9. Liquid Chromatography-Mass Spectrometry Analysis (LC-MS)

Individual polyphenolic acids and flavonoids were quantified in g *O. basilicum* cell suspension extracts using Waters Acquity UPLC I-class coupled with Xevo TQD MS (USA). Methanolic extract (5 mL) was shaken (at 4 °C for 30 min), followed by 5 min of gentle sonication (at 4 °C) and 10 min of centrifugation at 3000× *g*. The supernatant was collected and filtered using PVDF, 0.45 m (polyvinylidene difluoride, Thermo Fisher Scientific, Waltham, MA, USA). Compounds were measured using MS spectra by extracting the extracted ion current (EIC) to an accuracy of 40 parts per million (ppm), including using the appropriate standard curve regarding the molecular weight (MW) and chemical similarities. Diluted samples were injected (5 μL) using an auto-sampler injector with 2 μL: 1 mL n-hexane (LC grade) (Model Combi Pal, Varian). The gradient was as follows, using solvent A that contents of 1% formic acid in deionized water and acetonitrile as solvent B: solvent B 10–20% in 20 min, solvent B 20–25% in 10 min, solvent B 25–30% in 10 min), and gradient elution thereafter for 10 min with an adjusted flow of 8 mL/minute. For Waters Acquity UPLC I-class coupled with Xevo TQD MS (U.S.A), Acquity UPLC BEH C18 1.7 um–2.1 um × 100 mm column flow rate 0.5 mL min^−1^, the injection volume was 10 μL, Masslynix v4.1 software with Mass library (Milford, 017757 MA, USA), Argon as collision cell gas inlet 7 psi, and Nitrogen pressure 60 psi. The MS was configured to work in both negative and positive ion mode using an electrospray ionization (ESI) as an atmospheric pressure source. With a nebulizing flowrate of approximately 12 L/hour, the voltage of electrospray capillary was set to 3000 V and 300 °C of drying gas. Scan mode mass spectrometry data were acquired (mass range *m/z* 100–900). Compounds were measured using MS spectra by extracting the extracted ion chromatogram (EIC) with 30 ppm tolerance and comparing it to acceptable external calibration curves based on molecular weight and chemical similitude. Quantitative analyses were performed on three replicates for each condition, verifying assignments of molecules by Acquity UPLC I-class coupled with Xevo TQD MS analysis.

### 4.10. Evaluation of the Extracted Secondary Metabolites’ Contact-Insecticide and Antifeedant Efficacy against R. ferrugineus

The methanolic extract from the cell suspension of *O. basilicum* after 40 days and compounds (rosmarinic acid, nepetoidin B, ursolic acid, salvigenin, quercetin-3-O-rutinoside, rosmarinyl glucoside, chicoric acid, and isocitric acid) were prepared in acetone in serial concentrations 1, 10, 50, 100, 500, 1000, and 5000 µg/mL and, then, using 0.1% TritonX-100 to the constructed volume according to Darrag et al. [1] and Shukla et al. [68] with modifications. Serial concentrations (1, 10, 50, 100, 500, 1000, and 5000 µg/mL) of methanolic extract of cell suspension after 40 days and compounds (rosmarinic acid, nepetoidin B, ursolic acid, salvigenin, querce-tin-3-O-rutinoside, rosmarinyl glucoside, chicoric acid, and isocitric acid) were dissolved in acetone and diluted with 0.1% TritonX-100 according to Darrag et al. [1] and Shukla et al. [40]. The *R. ferrugineus* adult and larvae were acquired from an insect breeding lab at King Faisal University’s Date Palm Research Center of Excellence and grown utilizing sugarcane stem long pieces. The extracts activity against larvae was determined by using topical application method with storing larvae at 4–5 °C for 5 min. Previous serial concentrations of extract (10 µL) were applied to the dorsum of each larvae using a hand-operated micro-applicator (Burkard Manufacturing Co., Ltd., Hertfordshire, UK with a 50-ll micro-syringe (MS-N50; Ito Corp., Shizuoka, Japan). Each larva was feeding on 10 cm stem pieces of long sugarcane, with 5 larvae/box with three replicates. After topical administration, larval mortality was determined after 24, 48, 72, and 96 h to determine the LD_50_. Adults were tested for antifeedant effect determined using ten-centimeter-long sections of sugarcane stem divided into equal longitudinal halves. Pieces of long sections of sugarcane stem with area ~32.2 cm^2^ were dipped for ten seconds in the previous serial concentration of extract (10 mL) and, then, dried in room temperature air. Each item that had been treated was put in a plastic box. Each package received a replacement pair (male and female). Each treatment was replicated ten times. After 24, 48, 72, and 96 h, feeding observations were assayed.

### 4.11. Assessment of an O. basilicum Cell Suspension Extract and Pure Components on the Proteolytic Enzyme Activity (In Vitro) of R. ferrugineus Larvae

The Lowry technique was used to determine the protein concentrations [69]. Total proteolytic activity was determined in the fourth midgut instar larval (lab strain) homogenate of *R. ferrugineus* with azocasein in accordance with Darrag et al. [1] and Olga et al. [70]. Midgut larvae homogenates (10 larvae) were carefully extracted, dissected, and washed repeatedly with NaCl solution (0.9% (*w*/*v*), before being homogenized in 500 µL of an assay buffer (5 mM dithiothreitol (DTT), 50 mM HEPS (N-2-hydroxyethyl piperazin-N’-2-ehtanesulphonic acid), pH 8.0, and 0.1%, *v/v* Triton X-100). Using a Sigma 3k30 cooling centrifuge, reserve homogenates prepared in a previous stage were spun at 5000 rpm for 30 min. Estimates of protein content and total proteolytic enzyme activity were made using the supernatants. The supernatant (10 µL) was incubated at 37 °C for 20 min, and the volume was adjusted to 60 µL using an assay buffer (pH 8), before adding azocasien (200 µL, 2% (*w*/*v*)). In all instances, 10 μL enzyme samples, methanolic extract of *O. basilicum* cell suspension extract after 40 days, and pure chemicals (rosmarinic acid, nepetoidin B, ursolic acid, salvigenin, quercetin-3-O-rutinoside, rosmarinyl glucoside, chicoric acid, and isocitric acid) were incubated for 10 min. Following that, to activate the reaction, the substrate was introduced 20 min for Leupeptin, so the reaction was halted after 180 min at 37 °C with 300 μL of cool tri-chloroacetic acid (TCA) (10% (*v*/*v*). The mixture was centrifuged for 20 min at 5000 rpm (Sigma 3k30 cooling centrifuge). The supernatant was treated with 10 µL of NaOH (10 N), and the absorbance was determined at 450 nm using an ELISA plate reader, the total specific activity of proteolytic enzymes was approximated as OD450 mg^−1^·protein^−1^·h^−1^, and a blank solution sample devoid of enzyme solution was determined.

### 4.12. Assessment of an O. basilicum Cell Suspension Extract and Pure Components on Serine Proteinase Specific Activity (In Vitro) of R. ferrugineus Larvae

The specific activities of serine proteinase were determined using a rapid quick microplate assay with substrates in reaction mixtures (150 µL) including a serine protease assay buffer, according to Darrag et al. [1] and Olga et al. [70] with modifications.

The midguts were homogenized in the assay buffer (100 mM Tris-HCl, pH 8.1) and centrifuged for 30 min at 8000 rpm (Sigma 3K 30, rotors No. 12158) [1]. Each plate well received 10 µL of the enzyme for the trypsin-, chymotrypsin-, and elastase-like proteinases in the experiment (containing 40 µL of buffers). SAAPFρNA (100 mg/mL in DMF), BAρNA (100 mg/mL in DMSO), and SAAPLρNA (100 mg/mL in DMF) stock substrates were diluted in the assay buffer to 1 mg/mL, and substrate volume was 50 µL. The mixture was stirred at 37 °C for 15 min, then the reaction was stopped using 50 µL acetic acid (30%, *v*/*v*). The activity was quantified using an ELISA plate reader at a wavelength of 405 nm. As an empty well, an assay combination containing a denaturation enzyme was utilized instead of an active enzyme. Specific activities of proteinase were reported as OD/mg^−1^ protein^−1^ using substrates N-succinyl-ala-alapro-leucine ρ-nitroanilide (SAAPLρNA), Na-benzoyl-L-arginine ρ-nitroanilide (BAρNA), and N-succinyl-ala-ala-pro-phenylalanine ρ-nitroanilide (SAAPFρNA)).

### 4.13. Assessment of an O. basilicum Cell Suspension Extract and Pure Components on Metalloproteinase Specific Activity (In Vitro) of R. ferrugineus Larvae

A substrate azocasein assessed the metalloproteinases activity in the fourth midgut instar larval homogenate [1]. Ten larvae were carefully removed, dissected, and rinsed repeatedly with NaCl solution (0.9% (*w*/*v*). The midgut was homogenized using protease test solution (500 µL) containing 5 mM dithiothreitol (DTT), 50 mM HEPS (N-2-hydroxyelthyl piperazin-N’-2-ehtanesulphonic acid), an adjusted pH at 8.0, and Triton X-100 (0.1%, *v*/*v*). After homogenization, the homogenates were centrifuged at 5000 rpm (for 30 min) using a Sigma centrifuge 3k30, and the supernatant was utilized to determine enzyme activity and protein content. The supernatant per experiment (10 µL) was incubated for 20 min at 37 °C, and the volume was adjusted to 60 µL using assay buffer pH 8 before adding azocasein (200 µL, 2% (*w*/*v*)). In all instances, 10 µL enzyme samples were incubated for 10 min (except EDTA with 20 min), and EDTA was used as a blocker of metalloproteinase activity before being added to the specific substrate. The reaction was interrupted with 300 µL of trichloroacetic acid (TCA cold, 10% (*v*/*v*) and continued at 37 °C for 180 min. The reaction mixture was then centrifuged at 5000 rpm for 20 min (Sigma centrifuge 3k30), sodium hydroxide (10 µL of 10 N) was incorporated into the supernatant, then absorbance was determined using an ELISA plate reader at 450 nm. As a blank, an assay mixture devoid of the enzyme was devoted, the specific activity was determined using the OD450 mg^−1^ protein min^−1^ value, and a blank solution sample devoid of enzyme solution was determined.

### 4.14. Assessment of an O. basilicum Cell Suspension Extract and Pure Components on Cysteine Proteinase Specific Activity (In Vitro) of R. ferrugineus Larvae

The activity of cysteine proteinase was determined in the fourth midgut instar larval homogenate of *R. ferrugineus* using the substrate Z-Phe-Arg-MNA [1]. Ten larvae were carefully removed, dissected, and rinsed repeatedly with NaCl solution (0.9% (*w*/*v*). The midgut instar was homogenized in protease assay buffer (500 µL) [5 mM DTT, 50 mM HEPS (N-2-Hydroxyethyl piperazin-N’-2-ethanesulfonic acid), pH 8.0, and 0.1 percent Triton X-100]. The reaction mixture was centrifuged for 30 min at 5000 rpm (Sigma 3k30 cooling centrifuge). The supernatant was utilized to determine the overall activity of proteolytic enzymes and the protein content. The supernatant (10 µL) was incubated for 30 min at 37 °C, and the volume was adjusted to 60 µL using assay buffer (pH 8) before adding 100 µL of substrate (0.5 mM). Prior to adding the substrate, 10 µL enzyme samples were pre-incubated with iodoacetic acid for 10 min. After 60 min at 37 °C of mixture incubation, the reaction was halted with the addition of 1.5 mL mersalyl (5 mM), 0.02 mg/mL fast garnet reactive solution, and 2% Tween 20. The reaction was centrifuged (at 5000 rpm, 6 min), then the absorbance was determined (at 520 nm). The activity of cysteine proteinase was measured using an ELISA quick plate reader at the OD520/60 min/mg of midgut protein. As a blank, a test mixture devoid of enzyme was used. PMSF as general inhibitor of serine proteinase, TLCK as specific inhibitor of trypsin-like serine proteinase, TPCK as specific inhibitor of chymotrypsin-like serine proteinase, iodoacetic acid as specific inhibitor of cysteine proteinase, EDTA as specific inhibitor of metalloproteinase, and Leupeptin as general inhibitor of proteinase were added to reactions for demonstrate the specificity. The fourth homogenates of midgut larval were produced as reported before in the experiment for determining enzyme activity. As previously disclosed, the inhibitory experiment was performed using a microplate assay. A series of inhibitor concentrations was created to determine the inhibitor’s maximal inhibitory effect. Leupeptin concentrations of 0.01, 0.05, 0.1, and 1.0 mM, PMSF concentrations of 0.1, 1.0, 10, and 50 mM, TLCK and TPCK concentrations of 0.01, 0.05, 0.1, 1, 50, and 100 mM, EDTA concentrations of 0.1, 1, 10, 50, and 100 mM, and iodoacetic acid concentrations of 0.01, 0.05, 0.1, and 1 mM.

To learn more about the proteases found in homogenate preparations of *R. ferrugineus* fourth midgut larval instars, inhibitors from many different types of mechanisms were used. In all experiments for protease activity assay, 10 µL enzyme samples were pre-incubated for 10 min with extract or pure compounds (rosmarinic acid, nepetoidin B, ursolic acid, salvigenin, quercetin-3-O-rutinoside, rosmarinyl glucoside, chicoric acid, and isocitric acid) or inhibitors at concentrations of 1, 10, 50, 100, 500, 1000, and 5000 mg/L. After 24 h of treatment, the effect of treated larvae 1, 10, 50, 100, 500, 1000, and 5000 mg/L of extract or pure compounds on each enzyme activity was also evaluated in vivo. As a control, an assay combination devoid of inhibitors was utilized. The absorbance of the enzymes was determined using an ELISA plate quick reader at various wavelengths. The percentage activity of the control enzyme for each inhibitor was evaluated for each enzyme. Protease activity was assayed in vivo in the *R. ferrugineus* fourth midgut larval instars, which were treated with 1, 10, 50, 100, 500, 1000, and 5000 mg/L of extracts or compounds, measured as described above. Larvae were exposed for 24 h to leaf discs treated with extract and compounds.

### 4.15. Docking of Tested Compounds into Enzymes

Serine proteinase (PDB:2F7O) [71], metalloproteinase (PDB:1KAP) [72], and cysteine proteinase (PDB:3IOQ) [64] were obtained using protein data bank (PDB) and put into Molecular Operating Environment (MOE). The protein’s chemistry was adjusted for the missing hydrogen, and heteroatoms and crystallographic water molecules were deleted [73]. The Chem Draw professional 15 Builder module was used to generate compounds (rosmarinic acid, nepetoidin A, nepetoidin B, ursolic acid, salvigenin, quercetin-3-O-rutinoside, rosmarinic acid glucoside, chicoric acid, and isocitric acid). Prior to docking, the ligands were reduced, three-dimensional structures were constructed, duplicates were eliminated, and bonds were added. After initializing all default settings and getting the lowest energy structures after that, the ligands were permitted to just be elastic and positioned into enzyme model’s catalytic site cavity of the enzyme model manually. The docking was carried out with the MOE 2015.10, Chemical Computing Group Inc., Montreal, QC, Canada, using an induced-fit technique that assumes the receptor to be fixed and the ligand to be flexible [46]. The binding energy was determined using a field of full-force. A scoring system were used to determine the affinities between both the ligandcompounds and protein that estimated energy of non-covalent (free binding) interactions, using terminology pertaining to the molecular force field. The scoring functions and RMSD were calculated, and the optimal ligand-compound–protein interaction was reviewed and evaluated at conclusion of docking findings.

### 4.16. ADMET Screening

In silico ADMET screening was using ADMETLAB 2.0 to assess the toxicity risks of the tested pure compounds. Aqueous solubility, intestinal absorption, hydrogen bond acceptor (HBA), hydrogen bond donor (HBD), distribution coefficient P (LogP), solubility (LogS), plasma protein binding (PPB), blood-brain barrier (BBB), Cytochrome P450 (CYP450), and human hepatotoxicity (H-HT) were used to predict ADMET properties for each compound. The models utilized in this procedure to predict ADMET properties are derived from a number of empirical sources of data and are described in detail in the documentation.

### 4.17. Statistical Design

Finney [74] states that the statistical analysis and probit analysis were conducted using the SPSS 25.0 program (Statistical Package for Social Sciences, Armonk, NY, USA). Chemical components were determined by PCA using SPSS 25.0 program and presented using XLSTAT 2018.1. PCA is a clustering technique that is used to determine the relationships between samples. The score plot demonstrates reciprocal contrast, however, the loading plot clarifies cluster separation. The toxicity parameters, and results were reported as mean ± standard error. We did a mortality against dosage regression and converted the resultant median dose to an LC_50_ value (µg/mL). The range of LC_50_ was calculated with a 95 percent confidence interval using least-squares. We conducted a one-way analysis of variance on the enzyme activity data (ANOVA). The Student–Newman–Keuls (SNK) test was applied with separate means, and differences at *p* ≤ 0.05 were considered significant.

## 5. Conclusions

The results indicate that secondary metabolites isolated from *O. basilicum* extracts may be employed as a bio-insecticide against *R. ferrugineus*. The findings established a link between secondary metabolites and their usage, which might be linked to the polyphenolic and flavonoid chemicals contained in *O. basilicum*. The research is crucial for determining the efficacy and effects of such secondary metabolites. In silico ADMET and molecular docking analyses were performed, and the potential for developing eco-friendly bio-insecticides. Furthermore, large-scale generation of these secondary metabolites is possible utilizing cell suspension, a simple and clean process that opens the way to studying the produced and final formula for application.

## Figures and Tables

**Table 1:** Chemical contents of total phenolic, total flavonoid (TF), and condensed tannins (TCT) from cells from methanolic extracts of *O. basilicum* callus and cell suspensions infected by *V. dahliae*.

TF (mg of Quercetin/g DW)	TPC (mg of Gallic Acid/g DW)	Callus and Cell Suspension	TCT (mg of Cyanidins/g DW)
0.95 ^d^ ± 0.1324	9.78 ^d^ ± 0.1109	Callus without infection	0.24 ^d^ ± 0.0454
1.36 ^c^ ± 0.1562	14.85 ^c^ ± 0.1674	Callus with infection	0.38 ^c^ ± 0.0742
2.21 ^b^ ± 0.0413	19.23 ^b^ ± 0.1457	Cell suspension without infection	0.41 ^b^ ± 0.0316
3.97 ^a^ ± 0.0478	32.51 ^a^ ± 0.1904	Cell suspension with infection	0.74 ^a^ ± 0.1245

Mean ± Standard deviation (SD), n = 3, total phenolic content (TPC), total flavonoid (TF), total condensed tannins (TCT). Values followed by the same letter within a column are not significantly different (*p* ≤ 0.0005 ≡ significant for all values) according to Student–Newman–Keuls (SNK) test.

**Table 2:** Chemical composition of secondary metabolites, polyphenolic acids, and flavonoids detected from the cell suspensions of *O. basilicum* by Waters Acquity UPLC –I class coupled with Xevo TQD MS negative mode.

No.	Compounds Tentatively	RT (min)	RI (exp)	Formula	[M − H]^−^ (*m/z*)	Fragmentation Ions (*m/z*)	Content (µmol g^−1^ cell)
1	Tartaric acid	1.01	1249	C_4_H_5_O_6_	149.00	149, 141, 131, 113, 103, 87	0.18
2	Isocitric acid	2.54	1805.4	C_6_H_7_O_7_	191.0175	191, 173, 129, 111	1.58
3	Caffeic acid derivative (3TMS)	4.13	2155	C_18_H_32_O_4_Si_3_	359.70	396, 381, 359, 219, 191, 75	0.28
4	Caftaric acid (Caffeoyl-tartaric acid)	5.63	2701.3	C_13_H_12_O_9_	311.04	311, 179.03, 149.01, 135.04	0.38
5	Caffeic acid	6.28	1854.3	C_9_H_8_O_4_	179.03	179, 135	0.23
6	Fertaric acid	6.31	5191.1	C_14_H_14_O_9_	325.06	325, 193, 134	0.12
7	Salvianolic acid H/I	6.38	5237.8	C_27_H_22_O_12_	537.10	537, 493, 339, 313, 295, 197, 179	0.28
8	Salvianolic acid K	9.57	4556.9	C_27_H_24_O_13_	555.11	555, 537, 493, 295	0.22
9	Chicoric acid (dicaffeoyl-tartaric acid)	11.18	4552.3	C_22_H_18_O_12_	473.07	473, 311, 293, 179, 149	1.23
10	Lithospermic acid	11.31	4920.2	C_27_H_22_O_12_	537.10	537, 493, 356, 295	0.37
11	Dihydroquercetin 3-glucoside	11.46	4505.7	C_21_H_22_O_12_	456.10	467, 465, 313, 285, 259, 456, 175, 151	0.021
12	Quercetin-3-O-rutinoside (rutin)	11.53	4992.3	C_27_H_30_O_16_	611.16	611, 465, 449, 303	2.34
12	Rosmarinic acid	11.60	3504.5	C_18_H_16_O_8_	359.08	359, 197, 179, 161, 135, 117	14.2
13	Salvianolic acid E	12.69	4627.5	C_36_H_30_O_16_	717.15	717, 519, 475, 339	0.14
14	Salvianolic acid A	12.49	4585.8	C_26_H_22_O_10_	493.11	493, 313, 295, 185	0.42
15	Salvianolic acid B	12.61	5377.7	C_36_H_30_O_16_	717.15	717, 519, 321	0.54
16	Salvianolic acid F	17.94	4566.3	C_17_H_14_O_6_	313.07	313, 269	0.23
17	Cyanidin 3,3’-diglucoside	18.14	6158.2	C_27_H_31_O_16_	611.16	611, 287	0.021
18	Cyanidin 3-O-rutinoside (Cyaninoside)	18.27	5192.3	C_27_H_31_O_15_	595.17	595, 287	0.027
19	Salvigenin (5-Hydroxy-6,7,4′-trimethoxyflavone)	18.29	3121.7	C_18_H_16_O_6_	327.21	327, 311, 277, 215, 205, 116.9	2.51
20	Naringenin 7-0-glucoside	18.36	4081.3	C_21_H_22_Os_10_	434.4	435, 271, 151, 119	0.123
21	Apigenin 7-O-glucoside	18.45	4142.7	C_21_H_20_O_10_	432.4	432, 271, 171, 147, 119	0.078
22	Rosmarinic acid glucoside A	21.37	4023.4	C_24_H_26_O_13_	521.12	359, 197, 179, 161, 135	1.87
23	Rosmarinic acid glucoside B	25.07	4061.4	C_24_H_26_O_13_	521.12	359, 323, 197, 179, 161, 135	1.45
24	Nepetoidin A	25.53	4413.7	C_17_H_14_O_6_	314.29	335, 313, 161, 133	6.84
25	Nepetoidin B	25.67	4418.9	C_17_H_14_O_6_	314.29	335, 313, 269, 161, 133	5.72
26	Ursolic acid	26.06	3658.3	C_30_H_48_O_3_	456.7	591, 524, 523, 459, 455	4.91
27	Nepetoidin glucoside	27.99	4341	C_23_H_24_O_11_	475.12	475, 323, 313, 161, 151	1.23
28	Unknown	36.11	3697.2	ND	ND	ND	ND
29	Unknown	36.80	3751	ND	ND	ND	ND
30	Unknown	42.34	3508	ND	ND	ND	ND

The result was calculated as the mean of three replicates, *n* = 3, standard deviation (SD); the average relative abundances of each fragment ion are given in brackets; RT: retention time; [M−H]^−^: negative ion observed in a molecular ion (*m/z*: mass/charge); RI (exp): relative retention index determined. RI (exp) relative retention index from MS libraries (Wiley); National Institute of Standards and Technology (NIST).

**Table 3:** Mortality and probit analysis of *R. ferrugineus* adults and fourth larvae treated with *O. basilicum* extract and pure compounds.

Extract and Compounds	Adult				4th Larvae			
	LC_50_ (µg/mL)	Slope	Chi Square	*p*	LD_50_ (µg/Larvae)	Slope	Chi Square	*p*
*O. basilicum* extract	1238 (1038–1389)	2.64 ± 0.20	48.41	0.003	13.7 (12.9–15.6)	1.84 ± 0.26	43.42	0.001
Rosmarinic acid	1495 (1378–1504)	2.61 ± 0.22	42.63	0.007	12.4 (11.8–12.7)	1.94 ± 0.25	45.23	0.005
Nepetoidin B	1317 (1268–1346)	2.82 ± 0.20	43.29	0.004	11.9 (11.1–12.3)	1.97 ± 0.23	46.78	0.003
Ursolic acid	1167 (1038–1204)	2.85 ± 0.23	43.85	0.004	15.2 (14.2–15.9)	1.46 ± 0.28	41.21	0.003
Salvigenin	1189 (1049–1219)	2.87 ± 0.20	44.27	0.005	11.4 (10.4–11.8)	2.03 ± 0.22	47.54	0.004
Quercetin-3-O-rutinoside	1214 (1089–1234)	2.89 ± 0.24	42.36	0.003	16.9 (15.1–17.6)	1.42 ± 0.29	42.31	0.002
Rosmarinyl glucoside	1275 (1147–1315)	2.91 ± 0.21	40.85	0.003	17.6 (15.8–18.3)	1.40 ± 0.31	43.98	0.002
Isocitric acid	1826 (1712–1976)	2.12 ± 0.19	49.29	0.009	23.9 (21.1–24.6)	1.08 ± 0.30	42.36	0.008
Chicoric acid	1132 (1004–1198)	2.97 ± 0.21	44.17	0.003	10.23 (9.87–10.94)	2.07 ± 0.22	48.72	0.002

LC_50_: lethal concentration; LD_50_: lethal dose; CF: Confidence Limits; LC_50_ and LD_50_ with 95% CF. The data are given as the value ± standard error (SD) of five replicates for each concentration tested; *n* = 10.

**Table 4:** Docking scores of compounds inside the active sites of serine proteinase (PDB:2F7O), metalloproteinase (PDB:1KAP), and cysteine proteinase (PDB:3IOQ).

Compounds	Docking Score ΔG (kcal/mol)		
	Serine Proteinase	Cysteine Protease	Metalloprotease
Rosmarinic acid	−6.3212	−6.4056	−5.7938
Nepetoidin A	−5.9404	−5.3541	−6.0523
Nepetoidin B	−6.0265	−5.4078	−5.8765
Ursolic acid	−4.9541	−6.4766	−6.6226
Salvigenin	−6.2783	−5.3786	−6.2654
Quercetin-3-O-rutinoside	−8.1833	−6.5665	−5.6609
Rosmarinic acid glucoside	−7.7259	−6.5869	−6.0175
Isocitric acid	−4.3629	−4.1368	−4.4249
Chicoric acid	−6.3303	−5.8215	−6.8202

## Data Availability

Not applicable.

## Supplementary Materials

The following supporting information can be downloaded at: https://www.mdpi.com/article/10.3390/plants11081087/s1. Figure S1: LC-MS Chromatogram of *O. basilicum* cell suspension extracts. Figure S2: in vitro specific inhibitors against protease enzymes in *R. ferrugineus* larval instars; Leupeptin: general proteinase inhibitor; PMSF: a general inhibitor of serine proteinase (Elastase); TLCK: Trypsin inhibitor; TPCK: chymotrypsin inhibitor; EDTA: metalloprotease inhibitor; Iodoacetic acid: cysteine protease inhibitor. Figure S3: docking and ligand interactions of rosmarinic acid (A1, A2), nepetoidin A (B1, B2), nepetoidin B (C1, C2), quercetin-3-O-rutinoside (D1, D2), rosmarinyl glucoside (E1, E2), and isocitric acid (F1, F2) within the active sites of serine proteinase (PDB:2F7O). Figure S4: docking and ligand interactions of rosmarinic acid (A1, A2), nepetoidin A (B1, B2), nepetoidin B (C1, C2), salvigenin (D1, D2), chicoric acid (E1, E2), and isocitric acid (F1, F2) within the active sites of cysteine proteinase (PDB:3IOQ). Figure S5: docking and ligand interactions of rosmarinic acid (A1, A2), nepetoidin A (B1, B2), nepetoidin B (C1, C2), ursolic acid (D1, D2), salvigenin (E1, E2), and isocitric acid (F1, F2) within the active sites of metalloproteinase (PDB:1KAP). Table S1: ADMET analysis of rosmarinic acid, nepetoidin A, nepetoidin B, ursolic acid, salvigenin, quercetin-3-O-rutinoside, rosmarinyl glucoside, isocitric acid, and chicoric acid.
