# Supplementary material for: Secondary Metabolites in Basil, Bio-Insecticide, Inhibition Effect, and In Silico Molecular Docking against Proteolytic Enzymes of the Red Palm Weevil (Rhynchophorus ferrugineus)
Source: Plants (Basel). 2022 Apr 16;11(8):1087. doi: 10.3390/plants11081087 (PMC9027599; doi:10.3390/plants11081087)
Supplement: Supplementary file 1 [file plants-11-01087-s001.zip › plants-1677900-supplementary.pdf]

plants MDPI

Supplementary materials

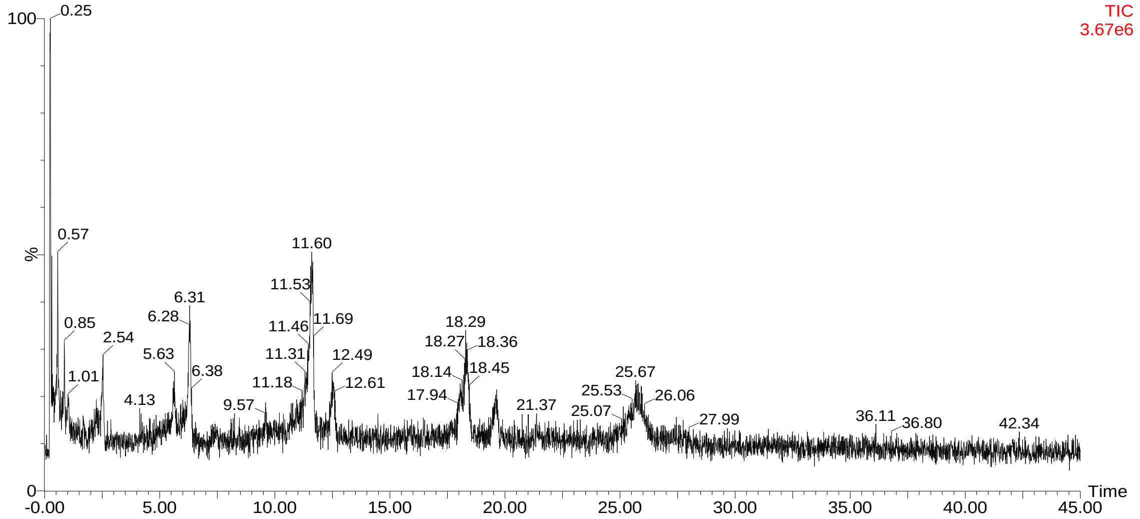

Figure S1. LC-MS Chromatogram of *O. basilicum* cell suspension extracts.

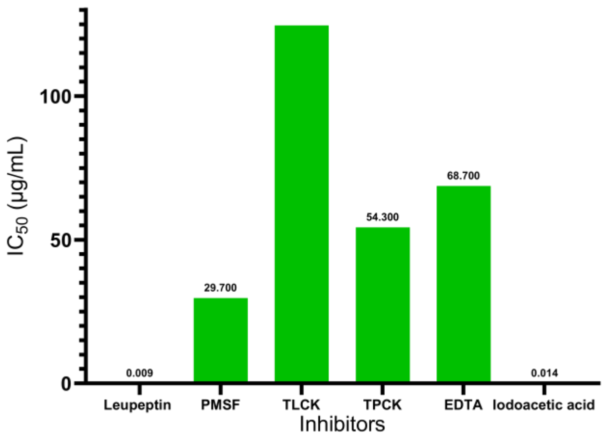

Figure S2. In vitro specific inhibitors against protease enzymes in *R. ferrugineus* larval instars; Leupeptin: general proteinase Inhibitor; PMSF: a general inhibitor of serine proteinase (Elastase); TLCK: Trypsin inhibitor; TPCK: chymotrypsin inhibitor; EDTA: metalloprotease inhibitor; Iodoacetic acid: cysteine protease inhibitor.

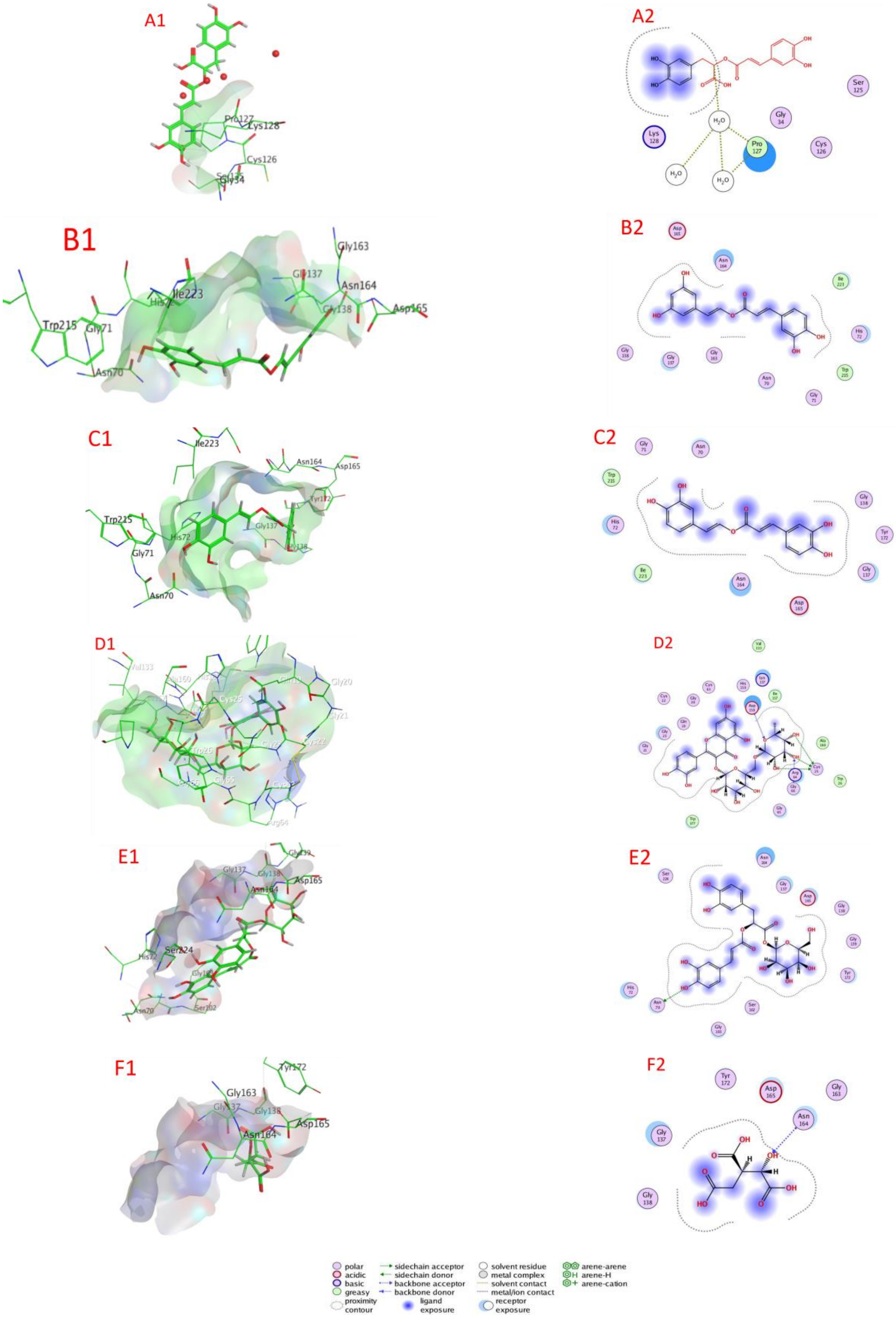

Figure S3. Docking and ligand Interactions of rosmarinic acid (A1, A2), nepetoidin A (B1, B2), nepetoidin B (C1, C2), quercetin-3-O-rutinoside (D1, D2), rosmarinyl glucoside (E1, E2), and isocitric acid (F1, F2) within the active sites of serine proteinase (PDB:2F7O).

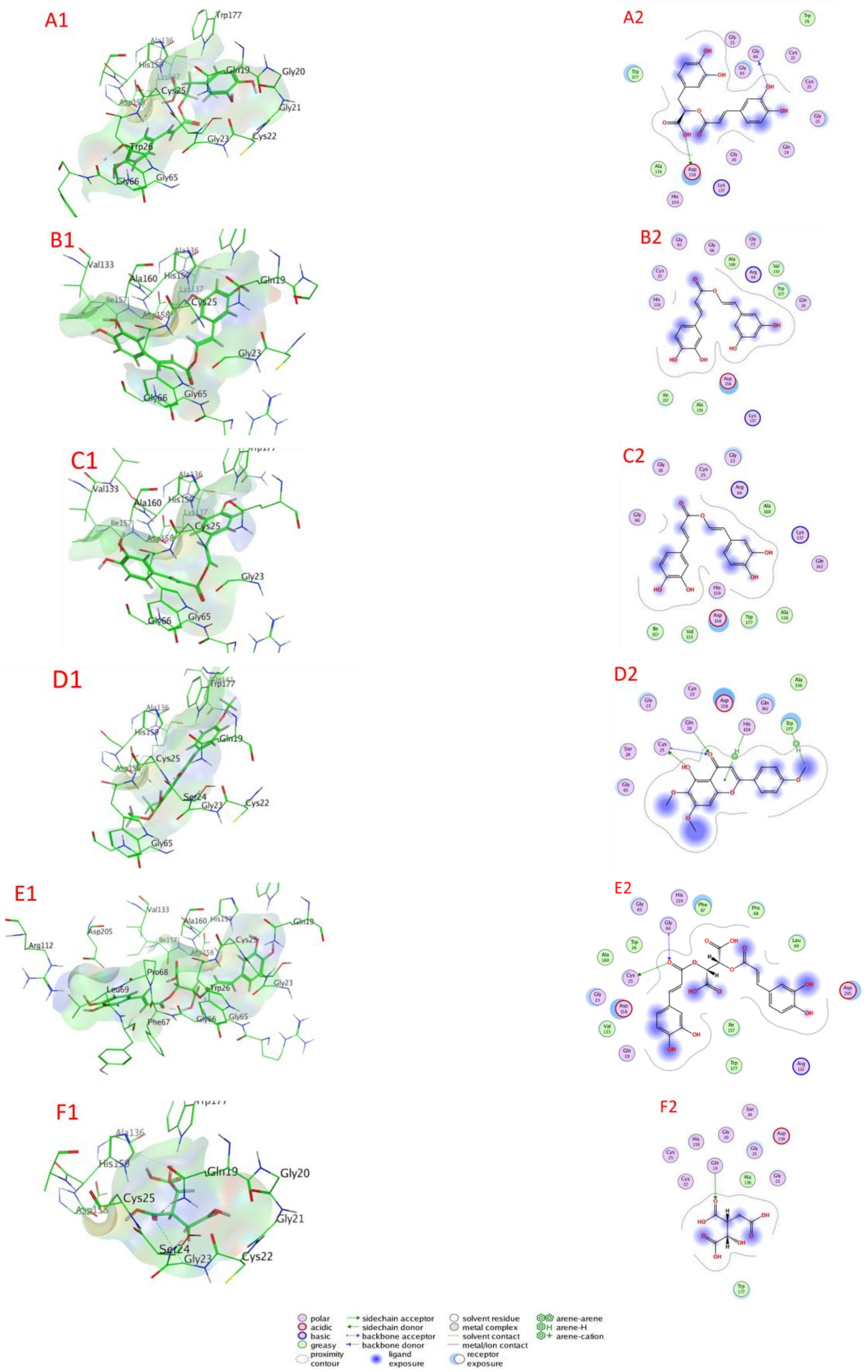

Figure S4. Docking and ligand Interactions of rosmarinic acid (A1, A2), nepetoidin A (B1, B2), nepetoidin B (C1, C2), salvigenin (D1, D2), chicoric acid (E1, E2), and isocitric acid (F1, F2) within the active sites of cysteine proteinase (PDB:3IOQ).

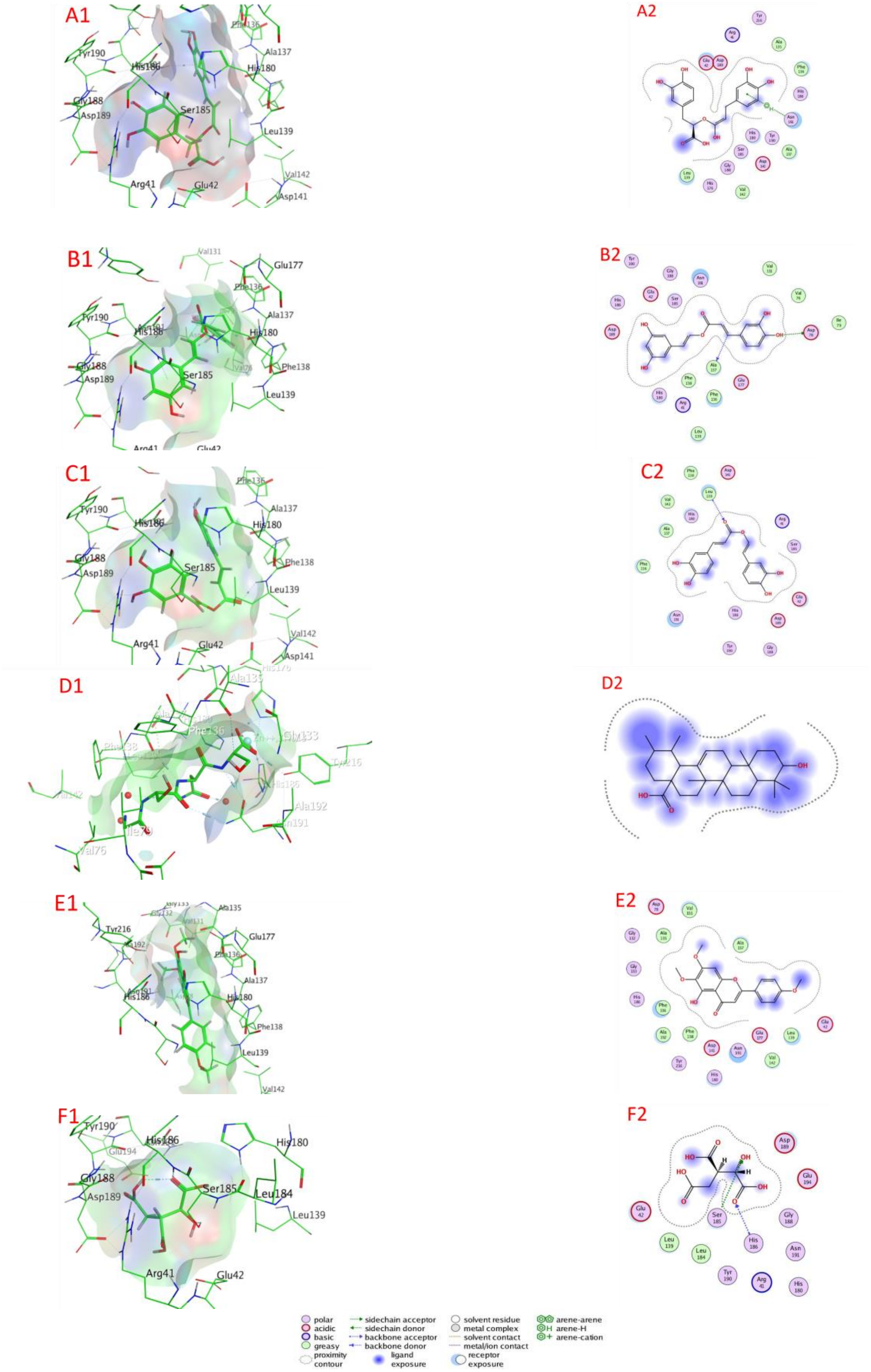

Figure S5. Docking and ligand Interactions of rosmarinic acid (A1, A2), nepetoidin A (B1, B2), nepetoidin B (C1, C2), ursolic acid (D1, D2), salvigenin (E1, E2), and isocitric acid (F1, F2) within the active sites of metalloproteinase (PDB:1KAP).

Table S1. ADMET analysis of rosmarinic acid, nepetoidin A, nepetoidin B, ursolic acid, salvigenin, quercetin-3-O-rutinoside, rosmarinyl glucoside, isocitric acid and chicoric acid.

Compounds	HBD	HBA	LogP	LogS (µg/mL)	BBB	PPB	CYP450 2D6 substrate	H-HT	TPSA
rosmarinic acid	5	7	1.761	96.535	++	+	+	+	144.52
Nepetoidin A	4	5	2.736	47.026	-	+	-	+	107.22
Nepetoidin B	4	6	2.736	47.026	-	+	-	+	107.22
Ursolic acid	2	2	7.09	0.187	++	+	-	---	57.53
Salvigenin	1	6	3.191	44.905	+	+	++	++	78.13
Rosmarinic acid glucoside	8	13	-0.98	473.208	---	+	+	-	223.67
Quercetin-3-O-rutinoside	10	16	-1.687	315.283	---	+	---	---	269.43
chicoric acid	6	10	1.228	97.526	+	+	-	-	208.12
Isocitric acid	4	7	-1.393	42518	+++	+	-	-	132.13

HBA: hydrogen bond acceptor; HBD: hydrogen bond donor; LogS: Solubility; LogP: Distribution Coefficient P; PPB: Plasma Protein Binding; BBB: Blood–Brain Barrier; H-HT: Human Hepatotoxicity. LogS: Optimal: higher than -4 log mol/L, LogS <10 µg/mL: Low solubility; LogS 10–60 µg/mL: Moderate solubility; LogS >60 µg/mL: High solubility; LogP: Optimal: 0< LogP <3, LogP <0: poor lipid bilayer permeability, LogP >3: poor aqueous solubility; BBB: BB ratio >=0.1: BBB+; BB ratio <0.1: BBB-; classification of models & probability 0~0.1 (---), 0.1~0.3 (--), 0.3~0.5 (-), 0.5~0.7 (+), 0.7~0.9 (++), 0.9~1 (+++).

.
